# Supplementary material for: Schizophrenia diagnosis based on diverse epoch size resting-state EEG using machine learning
Source: PeerJ Comput Sci. 2024 Aug 20;10:e2170. doi: 10.7717/peerj-cs.2170 (PMC11419632; doi:10.7717/peerj-cs.2170)
Supplement: Supplemental Information 1 [file peerj-cs-10-2170-s001.docx]

Table S1. One-Second Epoch Size Confusion Matrix Results.

| **Classifier** | **Feature Name** | **Classes Name** | | | **Predicted Class** | | | |
| --- | --- | --- | --- | --- | --- | --- | --- | --- |
| SVM | FFT | Actual Class | Sch | | 12634 | 382 | | |
|  |  |  | Healthy | | 403 | 15415 | | |
|  | ApEn | Actual Class | Sch | | 11311 | 1704 | | |
|  |  |  | Healthy | | 3149 | 10911 | | |
|  | ApEn+ Band-pass | Actual Class | Sch | | 11311 | 1704 | | |
|  |  |  | Healthy | | 3149 | 10911 | | |
|  | Shannon Entropy | Actual Class | Sch | | 13903 | 2760 | | |
|  |  |  | Healthy | | 807 | 12207 | | |
|  | Log Energy Entropy | Actual Class | Sch | | 15750 | 69 | | |
|  |  |  | Healthy | | 55 | 12960 | | |
|  | Kurtosis | Actual Class | Sch | | 11343 | 4476 | | |
|  |  |  | Healthy | | 3177 | 9838 | | |
| KNN | FFT | Actual Class | Sch | | 12303 | | 713 | |
|  |  |  | Healthy | | 715 | | 15103 | |
|  | ApEn | Actual Class | Sch | | 11040 | | 1975 | |
|  |  |  | Healthy | | 5219 | | 8841 | |
|  | ApEn+ Band-pass | Actual Class | Sch | | 11040 | | 1975 | |
|  |  |  | Healthy | | 5219 | | 8841 | |
|  | Shannon Entropy | Actual Class | Sch | | 15436 | | 1227 | |
|  |  |  | Healthy | | 1009 | | 12005 | |
|  | Log Energy Entropy | Actual Class | Sch | | 15633 | | 186 | |
|  |  |  | Healthy | | 116 | | 12899 | |
|  | Kurtosis | Actual Class | Sch | | 12365 | | 3454 | |
|  |  |  | Healthy | | 7036 | | 5979 | |
| QDA | FFT | Actual Class | Sch | | 12312 | | | 704 |
|  |  |  | Healthy | | 1186 | | | 14632 |
|  | ApEn | Actual Class | Sch | | 12810 | | | 205 |
|  |  |  | Healthy | | 6570 | | | 7490 |
|  | ApEn+ Band-pass | Actual Class | Sch | | 12810 | | | 205 |
|  |  |  | Healthy | | 6570 | | | 7490 |
|  | Shannon Entropy | Actual Class | Sch | | 12812 | | | 3851 |
|  |  |  | Healthy | | 1489 | | | 11525 |
|  | Log Energy Entropy | Actual Class | Sch | | 14988 | | | 831 |
|  |  |  | Healthy | | 55 | | | 12960 |
|  | Kurtosis | Actual Class | Sch | | 9358 | | | 6461 |
|  |  |  | Healthy | | 2591 | | | 10424 |
| EC | FFT | Actual Class | | Sch | 12635 | 381 | | |
|  |  |  |  | Healthy | 493 | 15326 | | |
|  | ApEn | Actual Class | | Sch | 11428 | 1587 | | |
|  |  |  |  | Healthy | 2514 | 13305 | | |
|  | ApEn+ Band-pass | Actual Class | | Sch | 12258 | 757 | | |
|  |  |  |  | Healthy | 4139 | 9921 | | |
|  | Shannon Entropy | Actual Class | | Sch | 12522 | 493 | | |
|  |  |  |  | Healthy | 776 | 15044 | | |
|  | Log Energy Entropy | Actual Class | | Sch | 15723 | 96 | | |
|  |  |  |  | Healthy | 57 | 12958 | | |
|  | Kurtosis | Actual Class | | Sch | 11208 | 4511 | | |
|  |  |  |  | Healthy | 3486 | 9529 | | |
