# Supplementary material for: Schizophrenia diagnosis based on diverse epoch size resting-state EEG using machine learning
Source: PeerJ Comput Sci. 2024 Aug 20;10:e2170. doi: 10.7717/peerj-cs.2170 (PMC11419632; doi:10.7717/peerj-cs.2170)
Supplement: Supplemental Information 2 [file peerj-cs-10-2170-s002.docx]

Table S2. One-Second Epoch Size Confusion Matrix Results with SNR

| **Classifier** | **Feature Name** | **Classes Name** | | | **Predicted Class** | | | |
| --- | --- | --- | --- | --- | --- | --- | --- | --- |
| SVM | FFT | Actual Class | Sch | | 293 | 15540 | | |
|  |  |  | Healthy | | 12737 | 292 | | |
|  | ApEn | Actual Class | Sch | | 5205 | 10628 | | |
|  |  |  | Healthy | | 10185 | 2844 | | |
|  | ApEn+ Band-pass | Actual Class | Sch | | 4598 | 11235 | | |
|  |  |  | Healthy | | 9781 | 3248 | | |
|  | Shannon Entropy+ Band-pass | Actual Class | Sch | | 2007 | 13826 | | |
|  |  |  | Healthy | | 12356 | 673 | | |
|  | Log Energy Entropy+ Band-pass | Actual Class | Sch | | 22 | 15811 | | |
|  |  |  | Healthy | | 12998 | 31 | | |
|  | Kurtosis+ Band-pass | Actual Class | Sch | | 4965 | 10868 | | |
|  |  |  | Healthy | | 8546 | 4483 | | |
| KNN | FFT | Actual Class | Sch | | 522 | | 15311 | |
|  |  |  | Healthy | | 12408 | | 621 | |
|  | ApEn | Actual Class | Sch | | 6052 | | 9781 | |
|  |  |  | Healthy | | 10527 | | 2502 | |
|  | ApEn+ Band-pass | Actual Class | Sch | | 6198 | | 9635 | |
|  |  |  | Healthy | | 10408 | | 2621 | |
|  | Shannon Entropy+ Band-pass | Actual Class | Sch | | 716 | | 15117 | |
|  |  |  | Healthy | | 12370 | | 659 | |
|  | Log Energy Entropy+ Band-pass | Actual Class | Sch | | 48 | | 15785 | |
|  |  |  | Healthy | | 12994 | | 35 | |
|  | Kurtosis+ Band-pass | Actual Class | Sch | | 4924 | | 10909 | |
|  |  |  | Healthy | | 7099 | | 5930 | |
| QDA | FFT | Actual Class | Sch | | 838 | | | 14995 |
|  |  |  | Healthy | | 12605 | | | 424 |
|  | ApEn | Actual Class | Sch | | 4617 | | | 11216 |
|  |  |  | Healthy | | 7671 | | | 5358 |
|  | ApEn+ Band-pass | Actual Class | Sch | | 6785 | | | 9048 |
|  |  |  | Healthy | | 11000 | | | 2029 |
|  | Shannon Entropy+ Band-pass | Actual Class | Sch | | 8607 | | | 7226 |
|  |  |  | Healthy | | 12792 | | | 237 |
|  | Log Energy Entropy+ Band-pass | Actual Class | Sch | | 21 | | | 15812 |
|  |  |  | Healthy | | 13010 | | | 19 |
|  | Kurtosis+ Band-pass | Actual Class | Sch | | 3211 | | | 12622 |
|  |  |  | Healthy | | 4551 | | | 8478 |
| EC | FFT | Actual Class | | Sch | 433 | 15400 | | |
|  |  |  |  | Healthy | 12705 | 324 | | |
|  | ApEn | Actual Class | | Sch | 5484 | 10349 | | |
|  |  |  |  | Healthy | 9622 | 3407 | | |
|  | ApEn+ Band-pass | Actual Class | | Sch | 4582 | 11251 | | |
|  |  |  |  | Healthy | 8295 | 4734 | | |
|  | Shannon Entropy+ Band-pass | Actual Class | | Sch | 95 | 15738 | | |
|  |  |  |  | Healthy | 12903 | 126 | | |
|  | Log Energy Entropy+ Band-pass | Actual Class | | Sch | 57 | 15775 | | |
|  |  |  |  | Healthy | 13000 | 30 | | |
|  | Kurtosis+ Band-pass | Actual Class | | Sch | 7148 | 8685 | | |
|  |  |  |  | Healthy | 9758 | 3271 | | |
