# Supplementary material for: Schizophrenia diagnosis based on diverse epoch size resting-state EEG using machine learning
Source: PeerJ Comput Sci. 2024 Aug 20;10:e2170. doi: 10.7717/peerj-cs.2170 (PMC11419632; doi:10.7717/peerj-cs.2170)
Supplement: Supplemental Information 3 [file peerj-cs-10-2170-s003.docx]

[INSERT Table S3. One-Second Epoch Size Confusion Matrix Results with Stretch.]

| **Classifier** | **Feature Name** | **Classes Name** | | | **Predicted Class** | | | |
| --- | --- | --- | --- | --- | --- | --- | --- | --- |
| SVM | FFT | Actual Class | Sch | | 471 | 17081 | | |
|  |  |  | Healthy | | 15240 | 395 | | |
|  | ApEn | Actual Class | Sch | | 1119 | 17879 | | |
|  |  |  | Healthy | | 14171 | 1464 | | |
|  | ApEn+ Band-pass | Actual Class | Sch | | 5147 | 13851 | | |
|  |  |  | Healthy | | 13722 | 1913 | | |
|  | Shannon Entropy+ Band-pass | Actual Class | Sch | | 3680 | 15318 | | |
|  |  |  | Healthy | | 14847 | 788 | | |
|  | Log Energy Entropy+ Band-pass | Actual Class | Sch | | 153 | 18845 | | |
|  |  |  | Healthy | | 15537 | 98 | | |
|  | Kurtosis+ Band-pass | Actual Class | Sch | | 6368 | 12630 | | |
|  |  |  | Healthy | | 12954 | 2681 | | |
| KNN | FFT | Actual Class | Sch | | 959 | | 16593 | |
|  |  |  | Healthy | | 14922 | | 713 | |
|  | ApEn | Actual Class | Sch | | 1193 | | 17805 | |
|  |  |  | Healthy | | 13618 | | 2017 | |
|  | ApEn+ Band-pass | Actual Class | Sch | | 9138 | | 9860 | |
|  |  |  | Healthy | | 14896 | | 739 | |
|  | Shannon Entropy+ Band-pass | Actual Class | Sch | | 1205 | | 17793 | |
|  |  |  | Healthy | | 14729 | | 906 | |
|  | Log Energy Entropy+ Band-pass | Actual Class | Sch | | 317 | | 18681 | |
|  |  |  | Healthy | | 15494 | | 141 | |
|  | Kurtosis+ Band-pass | Actual Class | Sch | | 5530 | | 13468 | |
|  |  |  | Healthy | | 10520 | | 5115 | |
| QDA | FFT | Actual Class | Sch | | 1588 | | | 15964 |
|  |  |  | Healthy | | 14914 | | | 721 |
|  | ApEn | Actual Class | Sch | | 4251 | | | 14747 |
|  |  |  | Healthy | | 13568 | | | 2067 |
|  | ApEn+ Band-pass | Actual Class | Sch | | 9670 | | | 9328 |
|  |  |  | Healthy | | 15413 | | | 222 |
|  | Shannon Entropy+ Band-pass | Actual Class | Sch | | 12322 | | | 6676 |
|  |  |  | Healthy | | 15386 | | | 249 |
|  | Log Energy Entropy+ Band-pass | Actual Class | Sch | | 1209 | | | 17789 |
|  |  |  | Healthy | | 15565 | | | 70 |
|  | Kurtosis+ Band-pass | Actual Class | Sch | | 17099 | | | 1899 |
|  |  |  | Healthy | | 15366 | | | 269 |
| EC | FFT | Actual Class | | Sch | 754 | 16798 | | |
|  |  |  |  | Healthy | 15226 | 409 | | |
|  | ApEn | Actual Class | | Sch | 1598 | 17400 | | |
|  |  |  |  | Healthy | 14134 | 1501 | | |
|  | ApEn+ Band-pass | Actual Class | | Sch | 6903 | 12095 | | |
|  |  |  |  | Healthy | 14631 | 1004 | | |
|  | Shannon Entropy+ Band-pass | Actual Class | | Sch | 495 | 18503 | | |
|  |  |  |  | Healthy | 15388 | 247 | | |
|  | Log Energy Entropy+ Band-pass | Actual Class | | Sch | 145 | 18853 | | |
|  |  |  |  | Healthy | 15581 | 54 | | |
|  | Kurtosis+ Band-pass | Actual Class | | Sch | 6507 | 12491 | | |
|  |  |  |  | Healthy | 13114 | 2521 | | |
