# Supplementary material for: Schizophrenia diagnosis based on diverse epoch size resting-state EEG using machine learning
Source: PeerJ Comput Sci. 2024 Aug 20;10:e2170. doi: 10.7717/peerj-cs.2170 (PMC11419632; doi:10.7717/peerj-cs.2170)
Supplement: Supplemental Information 4 [file peerj-cs-10-2170-s004.docx]

One-Second Epoch Size Confusion Matrix Results with 5 features Selection.

| **Feature Name** | **Classes Name** | | | **SVM** | | | |
| --- | --- | --- | --- | --- | --- | --- | --- |
|  |  |  |  | **Predicted Class** | | | |
| FFT | Actual Class | Sch | | 11912 | 798 | | |
|  |  | Healthy | | 1301 | 12980 | | |
| ApEn | Actual Class | Sch | | 10678 | 1982 | | |
|  |  | Healthy | | 5611 | 11041 | | |
| ApEn+ Band-pass | Actual Class | Sch | | 10140 | 1772 | | |
|  |  | Healthy | | 4761 | 10131 | | |
| Shannon Entropy+ Band-pass | Actual Class | Sch | | 12511 | 781 | | |
|  |  | Healthy | | 5712 | 4123 | | |
| Log Energy Entropy+ Band-pass | Actual Class | Sch | | 11980 | 120 | | |
|  |  | Healthy | | 411 | 11252 | | |
| Kurtosis+ Band-pass | Actual Class | Sch | | 9034 | 1882 | | |
|  |  | Healthy | | 4931 | 5113 | | |
| **Feature Name** | **Classes Name** | | | **KNN** | | | |
|  |  |  |  | **Predicted Class** | | | |
| FFT | Actual Class | Sch | | 11982 | | 673 | |
|  |  | Healthy | | 1829 | | 12981 | |
| ApEn | Actual Class | Sch | | 11001 | | 2981 | |
|  |  | Healthy | | 4981 | | 10134 | |
| ApEn+ Band-pass | Actual Class | Sch | | 11291 | | 1492 | |
|  |  | Healthy | | 4839 | | 2038 | |
| Shannon Entropy+ Band-pass | Actual Class | Sch | | 12678 | | 451 | |
|  |  | Healthy | | 2100 | | 12911 | |
| Log Energy Entropy+ Band-pass | Actual Class | Sch | | 5651 | | 21 | |
|  |  | Healthy | | 315 | | 5987 | |
| Kurtosis+ Band-pass | Actual Class | Sch | | 7980 | | 2010 | |
|  |  | Healthy | | 7869 | | 9126 | |
| **Feature Name** | **Classes Name** | | | **QDA** | | | |
|  |  |  |  | **Predicted Class** | | | |
| FFT | Actual Class | Sch | | 10182 | | | 881 |
|  |  | Healthy | | 3981 | | | 11612 |
| ApEn | Actual Class | Sch | | 12792 | | | 1811 |
|  |  | Healthy | | 5987 | | | 3412 |
| ApEn+ Band-pass | Actual Class | Sch | | 11655 | | | 253 |
|  |  | Healthy | | 7142 | | | 5381 |
| Shannon Entropy+ Band-pass | Actual Class | Sch | | 4894 | | | 831 |
|  |  | Healthy | | 6612 | | | 5612 |
| Log Energy Entropy+ Band-pass | Actual Class | Sch | | 11981 | | | 123 |
|  |  | Healthy | | 2012 | | | 12351 |
| Kurtosis+ Band-pass | Actual Class | Sch | | 12110 | | | 815 |
|  |  | Healthy | | 13105 | | | 1190 |
| **Feature Name** | **Classes Name** | | | **Ensemble** | | | |
|  |  |  |  | **Predicted Class** | | | |
| FFT | Actual Class | | Sch | 11781 | 859 | | |
|  |  |  | Healthy | 1129 | 10912 | | |
| ApEn | Actual Class | | Sch | 9861 | 2450 | | |
|  |  |  | Healthy | 5410 | 10820 | | |
| ApEn+ Band-pass | Actual Class | | Sch | 12654 | 967 | | |
|  |  |  | Healthy | 6113 | 7234 | | |
| Shannon Entropy+ Band-pass | Actual Class | | Sch | 12750 | 301 | | |
|  |  |  | Healthy | 566 | 14100 | | |
| Log Energy Entropy+ Band-pass | Actual Class | | Sch | 10751 | 121 | | |
|  |  |  | Healthy | 123 | 12912 | | |
| Kurtosis+ Band-pass | Actual Class | | Sch | 10911 | 978 | | |
|  |  |  | Healthy | 6541 | 5801 | | |
