# Supplementary material for: Schizophrenia diagnosis based on diverse epoch size resting-state EEG using machine learning
Source: PeerJ Comput Sci. 2024 Aug 20;10:e2170. doi: 10.7717/peerj-cs.2170 (PMC11419632; doi:10.7717/peerj-cs.2170)
Supplement: Supplemental Information 5 [file peerj-cs-10-2170-s005.docx]

One-Second Epoch Size Confusion Matrix Results with 8 features Selection**.**

| **Feature Name** | **Classes Name** | | | **SVM** | | | |
| --- | --- | --- | --- | --- | --- | --- | --- |
|  |  |  |  | **Predicted Class** | | | |
| FFT | Actual Class | Sch | | 11980 | 421 | | |
|  |  | Healthy | | 689 | 11157 | | |
| ApEn | Actual Class | Sch | | 9564 | 1456 | | |
|  |  | Healthy | | 2829 | 10040 | | |
| ApEn+ Band-pass | Actual Class | Sch | | 11987 | 2013 | | |
|  |  | Healthy | | 2151 | 1750 | | |
| Shannon Entropy+ Band-pass | Actual Class | Sch | | 11950 | 1921 | | |
|  |  | Healthy | | 3286 | 10011 | | |
| Log Energy Entropy+ Band-pass | Actual Class | Sch | | 11341 | 241 | | |
|  |  | Healthy | | 59 | 10451 | | |
| Kurtosis+ Band-pass | Actual Class | Sch | | 9890 | 2798 | | |
|  |  | Healthy | | 5899 | 9112 | | |
| **Feature Name** | **Classes Name** | | | **KNN** | | | |
|  |  |  |  | **Predicted Class** | | | |
| FFT | Actual Class | Sch | | 11521 | | 549 | |
|  |  | Healthy | | 563 | | 9165 | |
| ApEn | Actual Class | Sch | | 5718 | | 1180 | |
|  |  | Healthy | | 1452 | | 6183 | |
| ApEn+ Band-pass | Actual Class | Sch | | 6891 | | 1843 | |
|  |  | Healthy | | 3133 | | 5071 | |
| Shannon Entropy+ Band-pass | Actual Class | Sch | | 10203 | | 451 | |
|  |  | Healthy | | 667 | | 10512 | |
| Log Energy Entropy+ Band-pass | Actual Class | Sch | | 11560 | | 130 | |
|  |  | Healthy | | 122 | | 11912 | |
| Kurtosis+ Band-pass | Actual Class | Sch | | 8534 | | 4201 | |
|  |  | Healthy | | 5823 | | 9123 | |
| **Feature Name** | **Classes Name** | | | **QDA** | | | |
|  |  |  |  | **Predicted Class** | | | |
| FFT | Actual Class | Sch | | 10871 | | | 865 |
|  |  | Healthy | | 1419 | | | 11431 |
| ApEn | Actual Class | Sch | | 10120 | | | 1401 |
|  |  | Healthy | | 4152 | | | 2729 |
| ApEn+ Band-pass | Actual Class | Sch | | 6498 | | | 7354 |
|  |  | Healthy | | 810 | | | 11615 |
| Shannon Entropy+ Band-pass | Actual Class | Sch | | 6797 | | | 65 |
|  |  | Healthy | | 6412 | | | 2881 |
| Log Energy Entropy+ Band-pass | Actual Class | Sch | | 11198 | | | 54 |
|  |  | Healthy | | 1171 | | | 11192 |
| Kurtosis+ Band-pass | Actual Class | Sch | | 12289 | | | 126 |
|  |  | Healthy | | 13451 | | | 1201 |
| **Feature Name** | **Classes Name** | | | **Ensemble** | | | |
|  |  |  |  | **Predicted Class** | | | |
| FFT | Actual Class | | Sch | 6102 | 128 | | |
|  |  |  | Healthy | 412 | 5813 | | |
| ApEn | Actual Class | | Sch | 9687 | 1256 | | |
|  |  |  | Healthy | 2543 | 5647 | | |
| ApEn+ Band-pass | Actual Class | | Sch | 7432 | 1187 | | |
|  |  |  | Healthy | 1691 | 1611 | | |
| Shannon Entropy+ Band-pass | Actual Class | | Sch | 11982 | 142 | | |
|  |  |  | Healthy | 412 | 13641 | | |
| Log Energy Entropy+ Band-pass | Actual Class | | Sch | 12756 | 121 | | |
|  |  |  | Healthy | 131 | 11651 | | |
| Kurtosis+ Band-pass | Actual Class | | Sch | 10231 | 2432 | | |
|  |  |  | Healthy | 5413 | 7044 | | |
