# Supplementary material for: Schizophrenia diagnosis based on diverse epoch size resting-state EEG using machine learning
Source: PeerJ Comput Sci. 2024 Aug 20;10:e2170. doi: 10.7717/peerj-cs.2170 (PMC11419632; doi:10.7717/peerj-cs.2170)
Supplement: Supplemental Information 6 [file peerj-cs-10-2170-s006.docx]

Table S6. Two-Second Epoch Size Confusion Matrix Results

| **Classifier** | **Feature Name** | **Classes Name** | | | **Predicted Class** | | | |
| --- | --- | --- | --- | --- | --- | --- | --- | --- |
| SVM | FFT | Actual Class | Sch | | 5817 | 238 | | |
|  |  |  | Healthy | | 207 | 7706 | | |
|  | ApEn | Actual Class | Sch | | 5136 | 1374 | | |
|  |  |  | Healthy | | 1929 | 5984 | | |
|  | ApEn+ Band-pass | Actual Class | Sch | | 5581 | 929 | | |
|  |  |  | Healthy | | 1052 | 6861 | | |
|  | Shannon Entropy | Actual Class | Sch | | 1285 | 5225 | | |
|  |  |  | Healthy | | 362 | 7126 | | |
|  | Log Energy Entropy | Actual Class | Sch | | 6468 | 42 | | |
|  |  |  | Healthy | | 54 | 7859 | | |
|  | Kurtosis | Actual Class | Sch | | 4535 | 1975 | | |
|  |  |  | Healthy | | 1904 | 6085 | | |
| KNN | FFT | Actual Class | Sch | | 5616 | | 439 | |
|  |  |  | Healthy | | 376 | | 7537 | |
|  | ApEn | Actual Class | Sch | | 5935 | | 575 | |
|  |  |  | Healthy | | 3585 | | 4328 | |
|  | ApEn+ Band-pass | Actual Class | Sch | | 5557 | | 953 | |
|  |  |  | Healthy | | 1168 | | 6745 | |
|  | Shannon Entropy | Actual Class | Sch | | 5939 | | 571 | |
|  |  |  | Healthy | | 878 | | 6610 | |
|  | Log Energy Entropy | Actual Class | Sch | | 6438 | | 72 | |
|  |  |  | Healthy | | 112 | | 8701 | |
|  | Kurtosis | Actual Class | Sch | | 3412 | | 3098 | |
|  |  |  | Healthy | | 1652 | | 6337 | |
| QDA | FFT | Actual Class | Sch | | 5714 | | | 341 |
|  |  |  | Healthy | | 607 | | | 7306 |
|  | ApEn | Actual Class | Sch | | 6385 | | | 125 |
|  |  |  | Healthy | | 4052 | | | 3861 |
|  | ApEn+ Band-pass | Actual Class | Sch | | 5945 | | | 565 |
|  |  |  | Healthy | | 2676 | | | 5237 |
|  | Shannon Entropy | Actual Class | Sch | | 6344 | | | 166 |
|  |  |  | Healthy | | 4132 | | | 3356 |
|  | Log Energy Entropy | Actual Class | Sch | | 6485 | | | 25 |
|  |  |  | Healthy | | 407 | | | 7506 |
|  | Kurtosis | Actual Class | Sch | | 6379 | | | 131 |
|  |  |  | Healthy | | 6932 | | | 1057 |
| EC | FFT | Actual Class | | Sch | 5821 | 234 | | |
|  |  |  |  | Healthy | 273 | 7640 | | |
|  | ApEn | Actual Class | | Sch | 5709 | 801 | | |
|  |  |  |  | Healthy | 1299 | 6614 | | |
|  | ApEn+ Band-pass | Actual Class | | Sch | 5924 | 586 | | |
|  |  |  |  | Healthy | 2759 | 5154 | | |
|  | Shannon Entropy | Actual Class | | Sch | 6351 | 159 | | |
|  |  |  |  | Healthy | 269 | 7219 | | |
|  | Log Energy Entropy | Actual Class | | Sch | 6471 | 39 | | |
|  |  |  |  | Healthy | 74 | 7839 | | |
|  | Kurtosis | Actual Class | | Sch | 5260 | 1250 | | |
|  |  |  |  | Healthy | 2471 | 5518 | | |
