# Supplementary material for: Schizophrenia diagnosis based on diverse epoch size resting-state EEG using machine learning
Source: PeerJ Comput Sci. 2024 Aug 20;10:e2170. doi: 10.7717/peerj-cs.2170 (PMC11419632; doi:10.7717/peerj-cs.2170)
Supplement: Supplemental Information 7 [file peerj-cs-10-2170-s007.docx]

Table S7. Two-Second Epoch Size Confusion Matrix Results with SNR

| **Classifier** | **Feature Name** | **Classes Name** | | | **SVM** | | | |
| --- | --- | --- | --- | --- | --- | --- | --- | --- |
|  |  |  |  |  | **Predicted Class** | | | |
| SVM | FFT | Actual Class | Sch | | 282 | 7631 | | |
|  |  |  | Healthy | | 6217 | 293 | | |
|  | ApEn | Actual Class | Sch | | 1655 | 6255 | | |
|  |  |  | Healthy | | 4520 | 1990 | | |
|  | ApEn+Band-pass | Actual Class | Sch | | 1761 | 6152 | | |
|  |  |  | Healthy | | 5144 | 1366 | | |
|  | Shannon Entropy+ Band-pass | Actual Class | Sch | | 966 | 6947 | | |
|  |  |  | Healthy | | 6223 | 287 | | |
|  | Log Energy Entropy+ Band-pass | Actual Class | Sch | | 9 | 7904 | | |
|  |  |  | Healthy | | 6498 | 12 | | |
|  | Kurtosis+ Band-pass | Actual Class | Sch | | 2262 | 5651 | | |
|  |  |  | Healthy | | 4306 | 2204 | | |
| KNN | FFT | Actual Class | Sch | | 312 | | 7601 | |
|  |  |  | Healthy | | 6015 | | 495 | |
|  | ApEn | Actual Class | Sch | | 1770 | | 6143 | |
|  |  |  | Healthy | | 4412 | | 2098 | |
|  | ApEn+ Band-pass | Actual Class | Sch | | 2710 | | 5203 | |
|  |  |  | Healthy | | 5548 | | 962 | |
|  | Shannon Entropy+ Band-pass | Actual Class | Sch | | 256 | | 7657 | |
|  |  |  | Healthy | | 6261 | | 249 | |
|  | Log Energy Entropy+ Band-pass | Actual Class | Sch | | 17 | | 7896 | |
|  |  |  | Healthy | | 6495 | | 15 | |
|  | Kurtosis+ Band-pass | Actual Class | Sch | | 1521 | | 6392 | |
|  |  |  | Healthy | | 2503 | | 4007 | |
| QDA | FFT | Actual Class | Sch | | 455 | | | 7458 |
|  |  |  | Healthy | | 6187 | | | 323 |
|  | ApEn | Actual Class | Sch | | 949 | | | 6964 |
|  |  |  | Healthy | | 2318 | | | 4192 |
|  | ApEn+ Band-pass | Actual Class | Sch | | 3442 | | | 4471 |
|  |  |  | Healthy | | 5680 | | | 830 |
|  | Shannon Entropy+ Band-pass | Actual Class | Sch | | 4020 | | | 3893 |
|  |  |  | Healthy | | 6362 | | | 148 |
|  | Log Energy Entropy+ Band-pass | Actual Class | Sch | | 2 | | | 7911 |
|  |  |  | Healthy | | 6502 | | | 8 |
|  | Kurtosis+ Band-pass | Actual Class | Sch | | 462 | | | 7451 |
|  |  |  | Healthy | | 973 | | | 5537 |
| EC | FFT | Actual Class | | Sch | 258 | 7655 | | |
|  |  |  |  | Healthy | 6276 | 234 | | |
|  | ApEn | Actual Class | | Sch | 1975 | 5938 | | |
|  |  |  |  | Healthy | 4475 | 2035 | | |
|  | ApEn+ Band-pass | Actual Class | | Sch | 2474 | 5407 | | |
|  |  |  |  | Healthy | 5454 | 1056 | | |
|  | Shannon Entropy+ Band-pass | Actual Class | | Sch | 50 | 7863 | | |
|  |  |  |  | Healthy | 6459 | 51 | | |
|  | Log Energy Entropy+ Band-pass | Actual Class | | Sch | 9 | 7904 | | |
|  |  |  |  | Healthy | 6493 | 17 | | |
|  | Kurtosis+ Band-pass | Actual Class | | Sch | 3023 | 4890 | | |
|  |  |  |  | Healthy | 4713 | 1797 | | |
