# Supplementary material for: Schizophrenia diagnosis based on diverse epoch size resting-state EEG using machine learning
Source: PeerJ Comput Sci. 2024 Aug 20;10:e2170. doi: 10.7717/peerj-cs.2170 (PMC11419632; doi:10.7717/peerj-cs.2170)
Supplement: Supplemental Information 8 [file peerj-cs-10-2170-s008.docx]

Table S8. Two-Second Epoch Size Confusion Matrix Results with Stretch

| **Classifier** | **Feature Name** | **Classes Name** | | | **Predicted Class** | | | |
| --- | --- | --- | --- | --- | --- | --- | --- | --- |
| SVM | FFT | Actual Class | Sch | | 537 | 7376 | | |
|  |  |  | Healthy | | 5955 | 555 | | |
|  | ApEn | Actual Class | Sch | | 1124 | 6789 | | |
|  |  |  | Healthy | | 5386 | 1124 | | |
|  | ApEn+Band-pass | Actual Class | Sch | | 1841 | 6072 | | |
|  |  |  | Healthy | | 5849 | 661 | | |
|  | Shannon Entropy+ Band-pass | Actual Class | Sch | | 1628 | 7870 | | |
|  |  |  | Healthy | | 7438 | 379 | | |
|  | Log Energy Entropy+ Band-pass | Actual Class | Sch | | 41 | 9457 | | |
|  |  |  | Healthy | | 7789 | 28 | | |
|  | Kurtosis+ Band-pass | Actual Class | Sch | | 2784 | 5129 | | |
|  |  |  | Healthy | | 5237 | 1273 | | |
| KNN | FFT | Actual Class | Sch | | 607 | | 7306 | |
|  |  |  | Healthy | | 5954 | | 556 | |
|  | ApEn | Actual Class | Sch | | 1345 | | 6568 | |
|  |  |  | Healthy | | 55117 | | 993 | |
|  | ApEn+ Band-pass | Actual Class | Sch | | 3329 | | 4584 | |
|  |  |  | Healthy | | 6121 | | 389 | |
|  | Shannon Entropy+ Band-pass | Actual Class | Sch | | 512 | | 8986 | |
|  |  |  | Healthy | | 7384 | | 433 | |
|  | Log Energy Entropy+ Band-pass | Actual Class | Sch | | 71 | | 9427 | |
|  |  |  | Healthy | | 7767 | | 50 | |
|  | Kurtosis+ Band-pass | Actual Class | Sch | | 2379 | | 5534 | |
|  |  |  | Healthy | | 3807 | | 2730 | |
| QDA | FFT | Actual Class | Sch | | 1933 | | | 5980 |
|  |  |  | Healthy | | 5773 | | | 737 |
|  | ApEn | Actual Class | Sch | | 3480 | | | 4433 |
|  |  |  | Healthy | | 5326 | | | 1184 |
|  | ApEn+ Band-pass | Actual Class | Sch | | 3601 | | | 4312 |
|  |  |  | Healthy | | 6325 | | | 195 |
|  | Shannon Entropy+ Band-pass | Actual Class | Sch | | 5532 | | | 3966 |
|  |  |  | Healthy | | 7656 | | | 161 |
|  | Log Energy Entropy+ Band-pass | Actual Class | Sch | | 325 | | | 9173 |
|  |  |  | Healthy | | 7779 | | | 38 |
|  | Kurtosis+ Band-pass | Actual Class | Sch | | 6010 | | | 1903 |
|  |  |  | Healthy | | 5572 | | | 938 |
| EC | FFT | Actual Class | | Sch | 613 | 7300 | | |
|  |  |  |  | Healthy | 6050 | 460 | | |
|  | ApEn | Actual Class | | Sch | 1314 | 6599 | | |
|  |  |  |  | Healthy | 5316 | 1194 | | |
|  | ApEn+ Band-pass | Actual Class | | Sch | 1956 | 5957 | | |
|  |  |  |  | Healthy | 5847 | 663 | | |
|  | Shannon Entropy+ Band-pass | Actual Class | | Sch | 229 | 9269 | | |
|  |  |  |  | Healthy | 7677 | 140 | | |
|  | Log Energy Entropy+ Band-pass | Actual Class | | Sch | 39 | 9459 | | |
|  |  |  |  | Healthy | 7787 | 30 | | |
|  | Kurtosis+ Band-pass | Actual Class | | Sch | 2610 | 5303 | | |
|  |  |  |  | Healthy | 5245 | 1265 | | |
