# Supplementary material for: Schizophrenia diagnosis based on diverse epoch size resting-state EEG using machine learning
Source: PeerJ Comput Sci. 2024 Aug 20;10:e2170. doi: 10.7717/peerj-cs.2170 (PMC11419632; doi:10.7717/peerj-cs.2170)
Supplement: Supplemental Information 9 [file peerj-cs-10-2170-s009.docx]

Two-Second Epoch Size Confusion Matrix Results with 5 features Selection**.**

| **Feature Name** | **Classes Name** | | | **SVM** | | | |
| --- | --- | --- | --- | --- | --- | --- | --- |
|  |  |  |  | **Predicted Class** | | | |
| FFT | Actual Class | Sch | | 5194 | 533 | | |
|  |  | Healthy | | 679 | 7356 | | |
| ApEn | Actual Class | Sch | | 5101 | 1210 | | |
|  |  | Healthy | | 1100 | 6423 | | |
| ApEn_Entropy+Band-pass | Actual Class | Sch | | 4054 | 933 | | |
|  |  | Healthy | | 801 | 4211 | | |
| Shannon Entropy+ Band-pass | Actual Class | Sch | | 5152 | 465 | | |
|  |  | Healthy | | 3010 | 3976 | | |
| Log Energy Entropy+ Band-pass | Actual Class | Sch | | 4025 | 34 | | |
|  |  | Healthy | | 77 | 4041 | | |
| Kurtosis+ Band-pass | Actual Class | Sch | | 4085 | 1601 | | |
|  |  | Healthy | | 1958 | 4439 | | |
| **Feature Name** | **Classes Name** | | | **KNN** | | | |
|  |  |  |  | **Predicted Class** | | | |
| FFT | Actual Class | Sch | | 5467 | | 587 | |
|  |  | Healthy | | 765 | | 6859 | |
| ApEn | Actual Class | Sch | | 5912 | | 1121 | |
|  |  | Healthy | | 1225 | | 5198 | |
| ApEn_Entropy+ Band-pass | Actual Class | Sch | | 5911 | | 512 | |
|  |  | Healthy | | 3210 | | 4120 | |
| Shannon Entropy+ Band-pass | Actual Class | Sch | | 5748 | | 321 | |
|  |  | Healthy | | 411 | | 5439 | |
| Log Energy Entropy+ Band-pass | Actual Class | Sch | | 6321 | | 125 | |
|  |  | Healthy | | 132 | | 6534 | |
| Kurtosis+ Band-pass | Actual Class | Sch | | 3212 | | 57 | |
|  |  | Healthy | | 2197 | | 554 | |
| **Feature Name** | **Classes Name** | | | **QDA** | | | |
|  |  |  |  | **Predicted Class** | | | |
| FFT | Actual Class | Sch | | 5283 | | | 982 |
|  |  | Healthy | | 1899 | | | 5956 |
| ApEn | Actual Class | Sch | | 4586 | | | 1177 |
|  |  | Healthy | | 3320 | | | 4287 |
| ApEn_Entropy + Band-pass | Actual Class | Sch | | 5077 | | | 234 |
|  |  | Healthy | | 3154 | | | 4076 |
| Shannon Entropy+ Band-pass | Actual Class | Sch | | 3425 | | | 1287 |
|  |  | Healthy | | 2134 | | | 1486 |
| Log Energy Entropy+ Band-pass | Actual Class | Sch | | 4092 | | | 298 |
|  |  | Healthy | | 157 | | | 4211 |
| Kurtosis+ Band-pass | Actual Class | Sch | | 4895 | | | 824 |
|  |  | Healthy | | 5219 | | | 1321 |
| **Feature Name** | **Classes Name** | | | **Ensemble** | | | |
|  |  |  |  | **Predicted Class** | | | |
| FFT | Actual Class | | Sch | 6265 | 501 | | |
|  |  |  | Healthy | 128 | 675 | | |
| ApEn | Actual Class | | Sch | 5211 | 1300 | | |
|  |  |  | Healthy | 1123 | 5465 | | |
| ApEn_Entropy+ Band-pass | Actual Class | | Sch | 5166 | 395 | | |
|  |  |  | Healthy | 1765 | 4029 | | |
| Shannon Entropy+ Band-pass | Actual Class | | Sch | 6101 | 223 | | |
|  |  |  | Healthy | 109 | 5241 | | |
| Log Energy Entropy+ Band-pass | Actual Class | | Sch | 6236 | 122 | | |
|  |  |  | Healthy | 57 | 7532 | | |
| Kurtosis+ Band-pass | Actual Class | | Sch | 5243 | 1194 | | |
|  |  |  | Healthy | 3143 | 5976 | | |
