# Supplementary material for: Schizophrenia diagnosis based on diverse epoch size resting-state EEG using machine learning
Source: PeerJ Comput Sci. 2024 Aug 20;10:e2170. doi: 10.7717/peerj-cs.2170 (PMC11419632; doi:10.7717/peerj-cs.2170)
Supplement: Supplemental Information 10 [file peerj-cs-10-2170-s010.docx]

Two-Second Epoch Size Confusion Matrix Results with 8 features Selection**.**

| **Feature Name** | **Classes Name** | | | **SVM** | | | |
| --- | --- | --- | --- | --- | --- | --- | --- |
|  |  |  |  | **Predicted Class** | | | |
| FFT | Actual Class | Sch | | 4114 | 210 | | |
|  |  | Healthy | | 282 | 5064 | | |
| ApEn | Actual Class | Sch | | 4511 | 451 | | |
|  |  | Healthy | | 361 | 3573 | | |
| ApEn_Entropy+Band-pass | Actual Class | Sch | | 3913 | 389 | | |
|  |  | Healthy | | 1221 | 3241 | | |
| Shannon Entropy+ Band-pass | Actual Class | Sch | | 5288 | 321 | | |
|  |  | Healthy | | 1811 | 5074 | | |
| Log Energy Entropy+ Band-pass | Actual Class | Sch | | 6144 | 112 | | |
|  |  | Healthy | | 48 | 6575 | | |
| Kurtosis+ Band-pass | Actual Class | Sch | | 4723 | 1211 | | |
|  |  | Healthy | | 2743 | 4512 | | |
| **Feature Name** | **Classes Name** | | | **KNN** | | | |
|  |  |  |  | **Predicted Class** | | | |
| FFT | Actual Class | Sch | | 4495 | | 278 | |
|  |  | Healthy | | 312 | | 4171 | |
| ApEn | Actual Class | Sch | | 4671 | | 351 | |
|  |  | Healthy | | 412 | | 2712 | |
| ApEn_Entropy+ Band-pass | Actual Class | Sch | | 3412 | | 433 | |
|  |  | Healthy | | 1980 | | 4032 | |
| Shannon Entropy+ Band-pass | Actual Class | Sch | | 5120 | | 122 | |
|  |  | Healthy | | 197 | | 5111 | |
| Log Energy Entropy+ Band-pass | Actual Class | Sch | | 4421 | | 31 | |
|  |  | Healthy | | 47 | | 2371 | |
| Kurtosis+ Band-pass | Actual Class | Sch | | 4122 | | 2288 | |
|  |  | Healthy | | 2611 | | 4425 | |
| **Feature Name** | **Classes Name** | | | **QDA** | | | |
|  |  |  |  | **Predicted Class** | | | |
| FFT | Actual Class | Sch | | 4983 | | | 563 |
|  |  | Healthy | | 1001 | | | 5984 |
| ApEn | Actual Class | Sch | | 4944 | | | 1121 |
|  |  | Healthy | | 1135 | | | 2941 |
| ApEn_Entropy + Band-pass | Actual Class | Sch | | 6452 | | | 7181 |
|  |  | Healthy | | 222 | | | 14613 |
| Shannon Entropy+ Band-pass | Actual Class | Sch | | 5428 | | | 123 |
|  |  | Healthy | | 3542 | | | 4112 |
| Log Energy Entropy+ Band-pass | Actual Class | Sch | | 3442 | | | 43 |
|  |  | Healthy | | 91 | | | 2423 |
| Kurtosis+ Band-pass | Actual Class | Sch | | 5423 | | | 129 |
|  |  | Healthy | | 5997 | | | 1100 |
| **Feature Name** | **Classes Name** | | | **Ensemble** | | | |
|  |  |  |  | **Predicted Class** | | | |
| FFT | Actual Class | | Sch | 6032 | 211 | | |
|  |  |  | Healthy | 310 | 6111 | | |
| ApEn | Actual Class | | Sch | 5017 | 599 | | |
|  |  |  | Healthy | 659 | 5025 | | |
| ApEn_Entropy+ Band-pass | Actual Class | | Sch | 5381 | 1861 | | |
|  |  |  | Healthy | 1391 | 10242 | | |
| Shannon Entropy+ Band-pass | Actual Class | | Sch | 5022 | 36 | | |
|  |  |  | Healthy | 210 | 5112 | | |
| Log Energy Entropy+ Band-pass | Actual Class | | Sch | 3276 | 21 | | |
|  |  |  | Healthy | 33 | 2037 | | |
| Kurtosis+ Band-pass | Actual Class | | Sch | 5110 | 1232 | | |
|  |  |  | Healthy | 2355 | 5045 | | |
