# Supplementary material for: Schizophrenia diagnosis based on diverse epoch size resting-state EEG using machine learning
Source: PeerJ Comput Sci. 2024 Aug 20;10:e2170. doi: 10.7717/peerj-cs.2170 (PMC11419632; doi:10.7717/peerj-cs.2170)
Supplement: Supplemental Information 11 [file peerj-cs-10-2170-s011.docx]

Table S11. Five-Second Epoch Size Confusion Matrix Results

| **Classifier** | **Feature Name** | **Classes Name** | | **SVM** | | | |
| --- | --- | --- | --- | --- | --- | --- | --- |
|  |  |  |  | **Predicted Class** | | | |
| SVM | FFT | Actual Class | Sch | 2465 | 140 | | |
|  |  |  | Healthy | 131 | 3034 | | |
|  | ApEn | Actual Class | Sch | 2409 | 196 | | |
|  |  |  | Healthy | 1006 | 1942 | | |
|  | ApEn+ Band-pass | Actual Class | Sch | 2029 | 390 | | |
|  |  |  | Healthy | 460 | 2705 | | |
|  | Shannon Entropy | Actual Class | Sch | 2013 | 592 | | |
|  |  |  | Healthy | 261 | 2904 | | |
|  | Log Energy Entropy | Actual Class | Sch | 2582 | 23 | | |
|  |  |  | Healthy | 23 | 3142 | | |
|  | Kurtosis | Actual Class | Sch | 1852 | 753 | | |
|  |  |  | Healthy | 980 | 2185 | | |
| KNN | FFT | Actual Class | Sch | 2382 | | 223 | |
|  |  |  | Healthy | 217 | | 2948 | |
|  | ApEn | Actual Class | Sch | 2429 | | 176 | |
|  |  |  | Healthy | 1401 | | 1547 | |
|  | ApEn+ Band-pass | Actual Class | Sch | 1860 | | 559 | |
|  |  |  | Healthy | 361 | | 2804 | |
|  | Shannon Entropy | Actual Class | Sch | 2381 | | 224 | |
|  |  |  | Healthy | 395 | | 2770 | |
|  | Log Energy Entropy | Actual Class | Sch | 2573 | | 32 | |
|  |  |  | Healthy | 52 | | 3113 | |
|  | Kurtosis | Actual Class | Sch | 1894 | | 711 | |
|  |  |  | Healthy | 1101 | | 2064 | |
| QDA | FFT | Actual Class | Sch | 2450 | | | 155 |
|  |  |  | Healthy | 253 | | | 2912 |
|  | ApEn | Actual Class | Sch | 2521 | | | 84 |
|  |  |  | Healthy | 1397 | | | 1551 |
|  | ApEn+ Band-pass | Actual Class | Sch | 2186 | | | 233 |
|  |  |  | Healthy | 1056 | | | 2109 |
|  | Shannon Entropy | Actual Class | Sch | 2510 | | | 95 |
|  |  |  | Healthy | 1551 | | | 1614 |
|  | Log Energy Entropy | Actual Class | Sch | 2595 | | | 10 |
|  |  |  | Healthy | 123 | | | 3042 |
|  | Kurtosis | Actual Class | Sch | 2535 | | | 70 |
|  |  |  | Healthy | 2588 | | | 577 |
| EC | FFT | Actual Class | Sch | 2495 | | | 110 |
|  |  |  | Healthy | 174 | | | 2991 |
|  | ApEn | Actual Class | Sch | 2011 | | | 408 |
|  |  |  | Healthy | 532 | | | 2633 |
|  | ApEn+ Band-pass | Actual Class | Sch | 2388 | | | 217 |
|  |  |  | Healthy | 1018 | | | 1930 |
|  | Shannon Entropy | Actual Class | Sch | 2535 | | | 70 |
|  |  |  | Healthy | 120 | | | 304 |
|  | Log Energy Entropy | Actual Class | Sch | 2582 | | | 23 |
|  |  |  | Healthy | 45 | | | 3119 |
|  | Kurtosis | Actual Class | Sch | 2071 | | | 534 |
|  |  |  | Healthy | 1200 | | | 1965 |
