# Supplementary material for: Schizophrenia diagnosis based on diverse epoch size resting-state EEG using machine learning
Source: PeerJ Comput Sci. 2024 Aug 20;10:e2170. doi: 10.7717/peerj-cs.2170 (PMC11419632; doi:10.7717/peerj-cs.2170)
Supplement: Supplemental Information 12 [file peerj-cs-10-2170-s012.docx]

Table S12. Five-Second Epoch Size Confusion Matrix Results with SNR augmented method

| **Classifier** | **Feature Name** | **Classes Name** | | | **Predicted Class** | | | |
| --- | --- | --- | --- | --- | --- | --- | --- | --- |
| SVM | FFT | Actual Class | Sch | | 150 | 3015 | | |
|  |  |  | Healthy | | 2441 | 164 | | |
|  | ApEn | Actual Class | Sch | | 255 | 2940 | | |
|  |  |  | Healthy | | 2341 | 264 | | |
|  | ApEn+ Band-pass | Actual Class | Sch | | 375 | 2790 | | |
|  |  |  | Healthy | | 2237 | 368 | | |
|  | Shannon Entropy+ Band-pass | Actual Class | Sch | | 406 | 2759 | | |
|  |  |  | Healthy | | 2505 | 100 | | |
|  | Log Energy Entropy+ Band-pass | Actual Class | Sch | | 4 | 3161 | | |
|  |  |  | Healthy | | 2603 | 2 | | |
|  | Kurtosis+ Band-pass | Actual Class | Sch | | 818 | 2347 | | |
|  |  |  | Healthy | | 1790 | 815 | | |
| KNN | FFT | Actual Class | Sch | | 158 | | 3007 | |
|  |  |  | Healthy | | 2344 | | 261 | |
|  | ApEn | Actual Class | Sch | | 253 | | 2912 | |
|  |  |  | Healthy | | 2323 | | 282 | |
|  | ApEn+ Band-pass | Actual Class | Sch | | 476 | | 2689 | |
|  |  |  | Healthy | | 2156 | | 449 | |
|  | Shannon Entropy+ Band-pass | Actual Class | Sch | | 60 | | 3105 | |
|  |  |  | Healthy | | 2523 | | 82 | |
|  | Log Energy Entropy+ Band-pass | Actual Class | Sch | | 5 | | 3160 | |
|  |  |  | Healthy | | 2597 | | 8 | |
|  | Kurtosis+ Band-pass | Actual Class | Sch | | 964 | | 2201 | |
|  |  |  | Healthy | | 1504 | | 1101 | |
| QDA | FFT | Actual Class | Sch | | 204 | | | 2961 |
|  |  |  | Healthy | | 2429 | | | 176 |
|  | ApEn | Actual Class | Sch | | 320 | | | 2845 |
|  |  |  | Healthy | | 1655 | | | 950 |
|  | ApEn+ Band-pass | Actual Class | Sch | | 869 | | | 2296 |
|  |  |  | Healthy | | 2279 | | | 326 |
|  | Shannon Entropy+ Band-pass | Actual Class | Sch | | 1316 | | | 1849 |
|  |  |  | Healthy | | 2523 | | | 82 |
|  | Log Energy Entropy+ Band-pass | Actual Class | Sch | | 2 | | | 3163 |
|  |  |  | Healthy | | 2605 | | | 0 |
|  | Kurtosis+ Band-pass | Actual Class | Sch | | 638 | | | 2527 |
|  |  |  | Healthy | | 923 | | | 1682 |
| QDA | FFT | Actual Class | | Sch | 113 | 3052 | | |
|  |  |  |  | Healthy | 2460 | 145 | | |
|  | ApEn | Actual Class | | Sch | 244 | 2921 | | |
|  |  |  |  | Healthy | 2400 | 205 | | |
|  | ApEn+ Band-pass | Actual Class | | Sch | 432 | 2733 | | |
|  |  |  |  | Healthy | 2392 | 213 | | |
|  | Shannon Entropy+ Band-pass | Actual Class | | Sch | 12 | 3153 | | |
|  |  |  |  | Healthy | 2586 | 19 | | |
|  | Log Energy Entropy+ Band-pass | Actual Class | | Sch | 4 | 3161 | | |
|  |  |  |  | Healthy | 2599 | 6 | | |
|  | Kurtosis+ Band-pass | Actual Class | | Sch | 999 | 2166 | | |
|  |  |  |  | Healthy | 1990 | 615 | | |
