# Supplementary material for: Schizophrenia diagnosis based on diverse epoch size resting-state EEG using machine learning
Source: PeerJ Comput Sci. 2024 Aug 20;10:e2170. doi: 10.7717/peerj-cs.2170 (PMC11419632; doi:10.7717/peerj-cs.2170)
Supplement: Supplemental Information 13 [file peerj-cs-10-2170-s013.docx]

Table S13. Five-Second Epoch Size Confusion Matrix Results with Stretch signals.

| **Classifier** | **Feature Name** | **Classes Name** | | | **Predicted Class** | | | |
| --- | --- | --- | --- | --- | --- | --- | --- | --- |
| SVM | FFT | Actual Class | Sch | | 257 | 2908 | | |
|  |  |  | Healthy | | 2283 | 322 | | |
|  | ApEn | Actual Class | Sch | | 356 | 2809 | | |
|  |  |  | Healthy | | 2172 | 433 | | |
|  | ApEn+ Band-pass | Actual Class | Sch | | 387 | 2778 | | |
|  |  |  | Healthy | | 2340 | 265 | | |
|  | Shannon Entropy+ Band-pass | Actual Class | Sch | | 715 | 2450 | | |
|  |  |  | Healthy | | 2424 | 181 | | |
|  | Log Energy Entropy+ Band-pass | Actual Class | Sch | | 26 | 3139 | | |
|  |  |  | Healthy | | 2584 | 21 | | |
|  | Kurtosis+ Band-pass | Actual Class | Sch | | 823 | 2342 | | |
|  |  |  | Healthy | | 2027 | 578 | | |
| KNN | FFT | Actual Class | Sch | | 264 | | 2901 | |
|  |  |  | Healthy | | 2350 | | 255 | |
|  | ApEn | Actual Class | Sch | | 416 | | 2749 | |
|  |  |  | Healthy | | 2160 | | 445 | |
|  | ApEn+ Band-pass | Actual Class | Sch | | 563 | | 2602 | |
|  |  |  | Healthy | | 2204 | | 401 | |
|  | Shannon Entropy+ Band-pass | Actual Class | Sch | | 59 | | 3106 | |
|  |  |  | Healthy | | 2547 | | 58 | |
|  | Log Energy Entropy+ Band-pass | Actual Class | Sch | | 22 | | 3143 | |
|  |  |  | Healthy | | 2582 | | 23 | |
|  | Kurtosis+ Band-pass | Actual Class | Sch | | 915 | | 2250 | |
|  |  |  | Healthy | | 1791 | | 814 | |
| QDA | FFT | Actual Class | Sch | | 430 | | | 2735 |
|  |  |  | Healthy | | 2081 | | | 524 |
|  | ApEn | Actual Class | Sch | | 730 | | | 2435 |
|  |  |  | Healthy | | 1480 | | | 1125 |
|  | ApEn+ Band-pass | Actual Class | Sch | | 1507 | | | 1658 |
|  |  |  | Healthy | | 2459 | | | 146 |
|  | Shannon Entropy+ Band-pass | Actual Class | Sch | | 1622 | | | 1543 |
|  |  |  | Healthy | | 2532 | | | 73 |
|  | Log Energy Entropy+ Band-pass | Actual Class | Sch | | 417 | | | 2748 |
|  |  |  | Healthy | | 2569 | | | 36 |
|  | Kurtosis+ Band-pass | Actual Class | Sch | | 2157 | | | 1008 |
|  |  |  | Healthy | | 2275 | | | 330 |
| EC | FFT | Actual Class | | Sch | 240 | 2925 | | |
|  |  |  |  | Healthy | 2383 | 222 | | |
|  | ApEn | Actual Class | | Sch | 517 | 2648 | | |
|  |  |  |  | Healthy | 2140 | 465 | | |
|  | ApEn+ Band-pass | Actual Class | | Sch | 415 | 2750 | | |
|  |  |  |  | Healthy | 2426 | 179 | | |
|  | Shannon Entropy+ Band-pass | Actual Class | | Sch | 28 | 3137 | | |
|  |  |  |  | Healthy | 2545 | 60 | | |
|  | Log Energy Entropy+ Band-pass | Actual Class | | Sch | 17 | 3148 | | |
|  |  |  |  | Healthy | 2586 | 19 | | |
|  | Kurtosis+ Band-pass | Actual Class | | Sch | 845 | 2320 | | |
|  |  |  |  | Healthy | 2199 | 406 | | |
