# Supplementary material for: Schizophrenia diagnosis based on diverse epoch size resting-state EEG using machine learning
Source: PeerJ Comput Sci. 2024 Aug 20;10:e2170. doi: 10.7717/peerj-cs.2170 (PMC11419632; doi:10.7717/peerj-cs.2170)
Supplement: Supplemental Information 14 — Five-Second Epoch Size Confusion Matrix Results with 5 electrodes [file peerj-cs-10-2170-s014.docx]

Five-Second Epoch Size Confusion Matrix Results with 5 features Selection**.**

| **Feature Name** | **Classes Name** | | | **SVM** | | | |
| --- | --- | --- | --- | --- | --- | --- | --- |
|  |  |  |  | **Predicted Class** | | | |
| FFT | Actual Class | Sch | | 1283 | 121 | | |
|  |  | Healthy | | 230 | 1462 | | |
| ApEn | Actual Class | Sch | | 2101 | 235 | | |
|  |  | Healthy | | 356 | 1481 | | |
| ApEn_Entropy+ Band-pass | Actual Class | Sch | | 2114 | 190 | | |
|  |  | Healthy | | 290 | 1156 | | |
| Shannon Entropy+ Band-pass | Actual Class | Sch | | 2151 | 133 | | |
|  |  | Healthy | | 845 | 1122 | | |
| Log Energy Entropy+ Band-pass | Actual Class | Sch | | 4966 | 40 | | |
|  |  | Healthy | | 98 | 4987 | | |
| Kurtosis+ Band-pass | Actual Class | Sch | | 1290 | 403 | | |
|  |  | Healthy | | 908 | 1990 | | |
| **Feature Name** | **Classes Name** | | | **KNN** | | | |
|  |  |  |  | **Predicted Class** | | | |
| FFT | Actual Class | Sch | | 2161 | | 213 | |
|  |  | Healthy | | 230 | | 2235 | |
| ApEn | Actual Class | Sch | | 2011 | | 430 | |
|  |  | Healthy | | 390 | | 2583 | |
| ApEn_Entropy+ Band-pass | Actual Class | Sch | | 1824 | | 394 | |
|  |  | Healthy | | 413 | | 2111 | |
| Shannon Entropy+ Band-pass | Actual Class | Sch | | 2276 | | 67 | |
|  |  | Healthy | | 171 | | 2390 | |
| Log Energy Entropy+ Band-pass | Actual Class | Sch | | 1987 | | 11 | |
|  |  | Healthy | | 81 | | 2109 | |
| Kurtosis+ Band-pass | Actual Class | Sch | | 1543 | | 632 | |
|  |  | Healthy | | 1033 | | 1567 | |
| **Feature Name** | **Classes Name** | | | **QDA** | | | |
|  |  |  |  | **Predicted Class** | | | |
| FFT | Actual Class | Sch | | 1879 | | | 313 |
|  |  | Healthy | | 390 | | | 1421 |
| ApEn | Actual Class | Sch | | 1092 | | | 1232 |
|  |  | Healthy | | 520 | | | 2199 |
| ApEn_Entropy+ Band-pass | Actual Class | Sch | | 1113 | | | 43 |
|  |  | Healthy | | 499 | | | 213 |
| Shannon Entropy+ Band-pass | Actual Class | Sch | | 2141 | | | 322 |
|  |  | Healthy | | 975 | | | 1231 |
| Log Energy Entropy+ Band-pass | Actual Class | Sch | | 1212 | | | 21 |
|  |  | Healthy | | 190 | | | 1119 |
| Kurtosis+ Band-pass | Actual Class | Sch | | 1987 | | | 342 |
|  |  | Healthy | | 1620 | | | 717 |
| **Feature Name** | **Classes Name** | | | **Ensemble** | | | |
|  |  |  |  | **Predicted Class** | | | |
| FFT | Actual Class | | Sch | 1181 | 162 | | |
|  |  |  | Healthy | 170 | 2113 | | |
| ApEn | Actual Class | | Sch | 1111 | 324 | | |
|  |  |  | Healthy | 423 | 2003 | | |
| ApEn_Entropy+ Band-pass | Actual Class | | Sch | 2133 | 201 | | |
|  |  |  | Healthy | 415 | 2311 | | |
| Shannon Entropy+ Band-pass | Actual Class | | Sch | 2341 | 36 | | |
|  |  |  | Healthy | 43 | 1189 | | |
| Log Energy Entropy+ Band-pass | Actual Class | | Sch | 2132 | 26 | | |
|  |  |  | Healthy | 21 | 2456 | | |
| Kurtosis+ Band-pass | Actual Class | | Sch | 1762 | 412 | | |
|  |  |  | Healthy | 883 | 1751 | | |
