# Supplementary material for: Schizophrenia diagnosis based on diverse epoch size resting-state EEG using machine learning
Source: PeerJ Comput Sci. 2024 Aug 20;10:e2170. doi: 10.7717/peerj-cs.2170 (PMC11419632; doi:10.7717/peerj-cs.2170)
Supplement: Supplemental Information 15 [file peerj-cs-10-2170-s015.docx]

Five-Second Epoch Size Confusion Matrix Results with 8 features Selection**.**

| **Feature Name** | **Classes Name** | | | **SVM** | | | |
| --- | --- | --- | --- | --- | --- | --- | --- |
|  |  |  |  | **Predicted Class** | | | |
| FFT | Actual Class | Sch | | 2121 | 199 | | |
|  |  | Healthy | | 132 | 1981 | | |
| ApEn | Actual Class | Sch | | 1265 | 116 | | |
|  |  | Healthy | | 88 | 1675 | | |
| ApEn_Entropy+ Band-pass | Actual Class | Sch | | 1879 | 189 | | |
|  |  | Healthy | | 231 | 1989 | | |
| Shannon Entropy+ Band-pass | Actual Class | Sch | | 1768 | 123 | | |
|  |  | Healthy | | 614 | 1799 | | |
| Log Energy Entropy+ Band-pass | Actual Class | Sch | | 1354 | 11 | | |
|  |  | Healthy | | 33 | 1678 | | |
| Kurtosis+ Band-pass | Actual Class | Sch | | 1981 | 476 | | |
|  |  | Healthy | | 763 | 1493 | | |
| **Feature Name** | **Classes Name** | | | **KNN** | | | |
|  |  |  |  | **Predicted Class** | | | |
| FFT | Actual Class | Sch | | 5098 | | 449 | |
|  |  | Healthy | | 391 | | 6111 | |
| ApEn | Actual Class | Sch | | 2301 | | 177 | |
|  |  | Healthy | | 137 | | 2101 | |
| ApEn_Entropy+ Band-pass | Actual Class | Sch | | 2150 | | 211 | |
|  |  | Healthy | | 428 | | 2181 | |
| Shannon Entropy+ Band-pass | Actual Class | Sch | | 2154 | | 45 | |
|  |  | Healthy | | 111 | | 2764 | |
| Log Energy Entropy+ Band-pass | Actual Class | Sch | | 1021 | | 7 | |
|  |  | Healthy | | 14 | | 1036 | |
| Kurtosis+ Band-pass | Actual Class | Sch | | 1611 | | 798 | |
|  |  | Healthy | | 880 | | 2149 | |
| **Feature Name** | **Classes Name** | | | **QDA** | | | |
|  |  |  |  | **Predicted Class** | | | |
| FFT | Actual Class | Sch | | 2132 | | | 331 |
|  |  | Healthy | | 215 | | | 2161 |
| ApEn | Actual Class | Sch | | 1355 | | | 461 |
|  |  | Healthy | | 215 | | | 1172 |
| ApEn_Entropy+ Band-pass | Actual Class | Sch | | 2782 | | | 133 |
|  |  | Healthy | | 1621 | | | 2721 |
| Shannon Entropy+ Band-pass | Actual Class | Sch | | 2154 | | | 69 |
|  |  | Healthy | | 1422 | | | 1167 |
| Log Energy Entropy+ Band-pass | Actual Class | Sch | | 2599 | | | 6 |
|  |  | Healthy | | 6 | | | 1589 |
| Kurtosis+ Band-pass | Actual Class | Sch | | 2132 | | | 298 |
|  |  | Healthy | | 2211 | | | 959 |
| **Feature Name** | **Classes Name** | | | **Ensemble** | | | |
|  |  |  |  | **Predicted Class** | | | |
| FFT | Actual Class | | Sch | 1376 | 109 | | |
|  |  |  | Healthy | 78 | 2091 | | |
| ApEn | Actual Class | | Sch | 2113 | 123 | | |
|  |  |  | Healthy | 209 | 1190 | | |
| ApEn_Entropy+ Band-pass | Actual Class | | Sch | 2421 | 98 | | |
|  |  |  | Healthy | 255 | 2128 | | |
| Shannon Entropy+ Band-pass | Actual Class | | Sch | 2433 | 90 | | |
|  |  |  | Healthy | 58 | 2989 | | |
| Log Energy Entropy+ Band-pass | Actual Class | | Sch | 1789 | 11 | | |
|  |  |  | Healthy | 34 | 2143 | | |
| Kurtosis+ Band-pass | Actual Class | | Sch | 1176 | 256 | | |
|  |  |  | Healthy | 628 | 1798 | | |
