# Supplementary figures and images for: Schizophrenia diagnosis based on diverse epoch size resting-state EEG using machine learning
Source: PeerJ Comput Sci. 2024 Aug 20;10:e2170. doi: 10.7717/peerj-cs.2170 (PMC11419632; doi:10.7717/peerj-cs.2170)

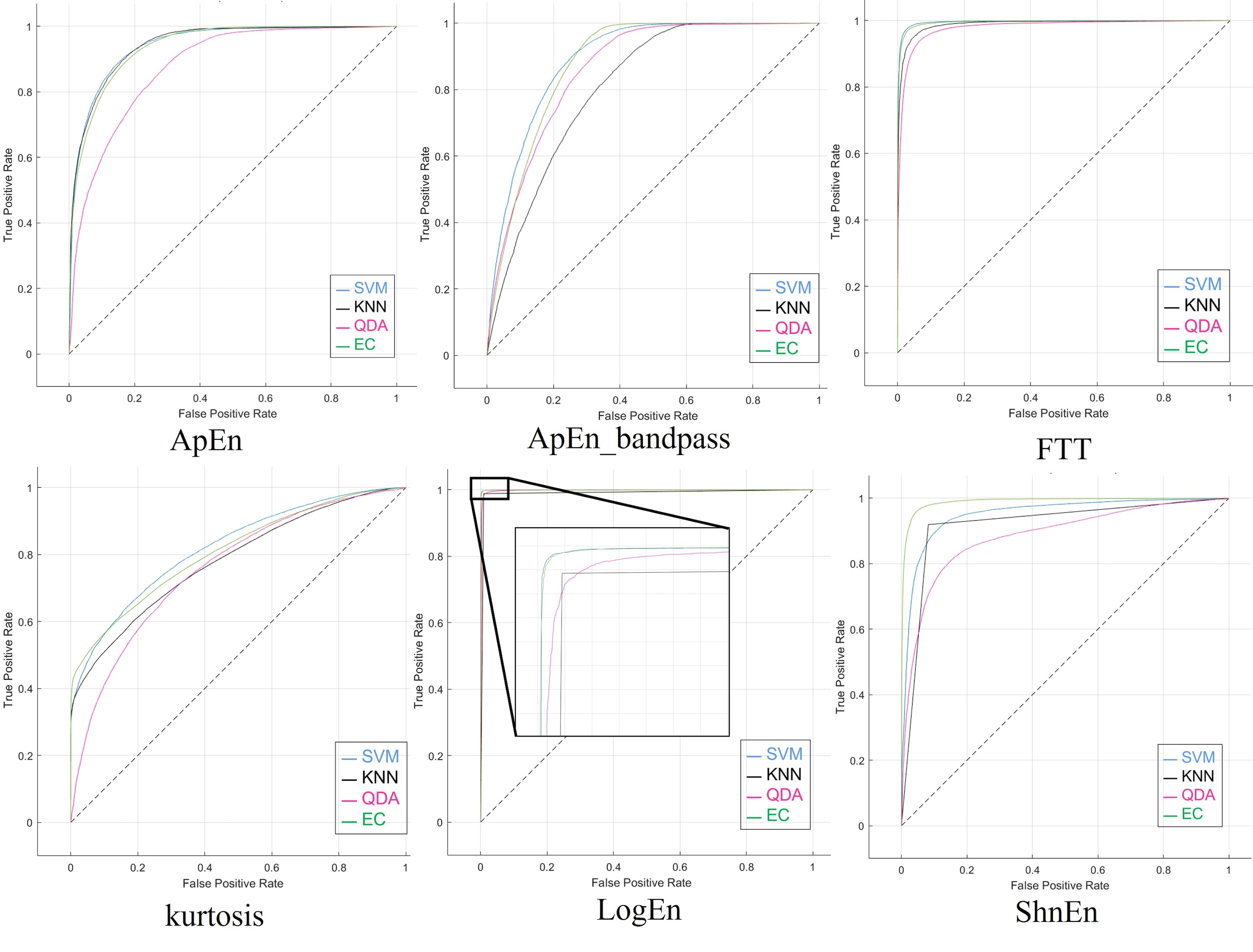

Supplement: Supplemental Information 16 [file peerj-cs-10-2170-s016.jpg]

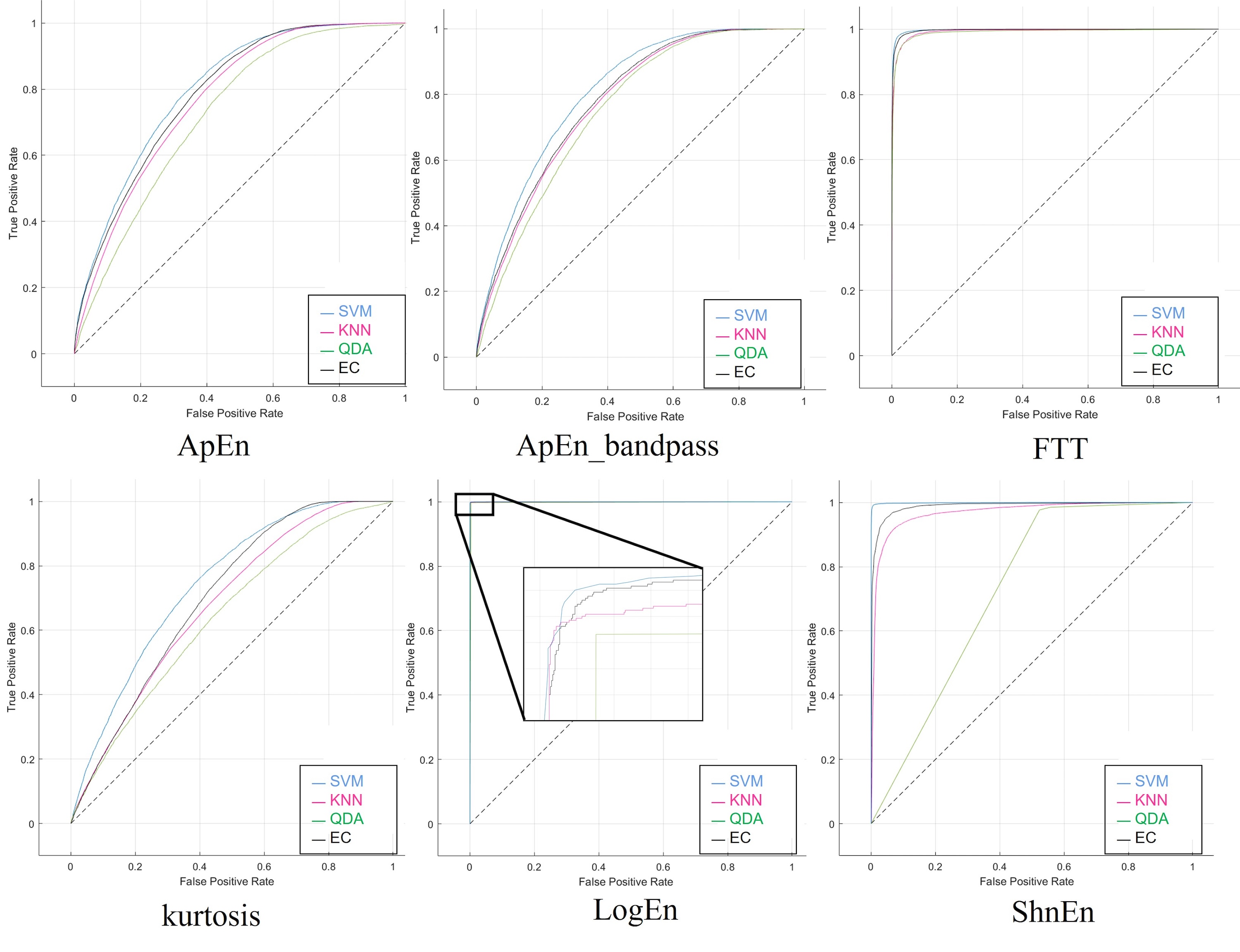

Supplement: Supplemental Information 17 [file peerj-cs-10-2170-s017.jpg]

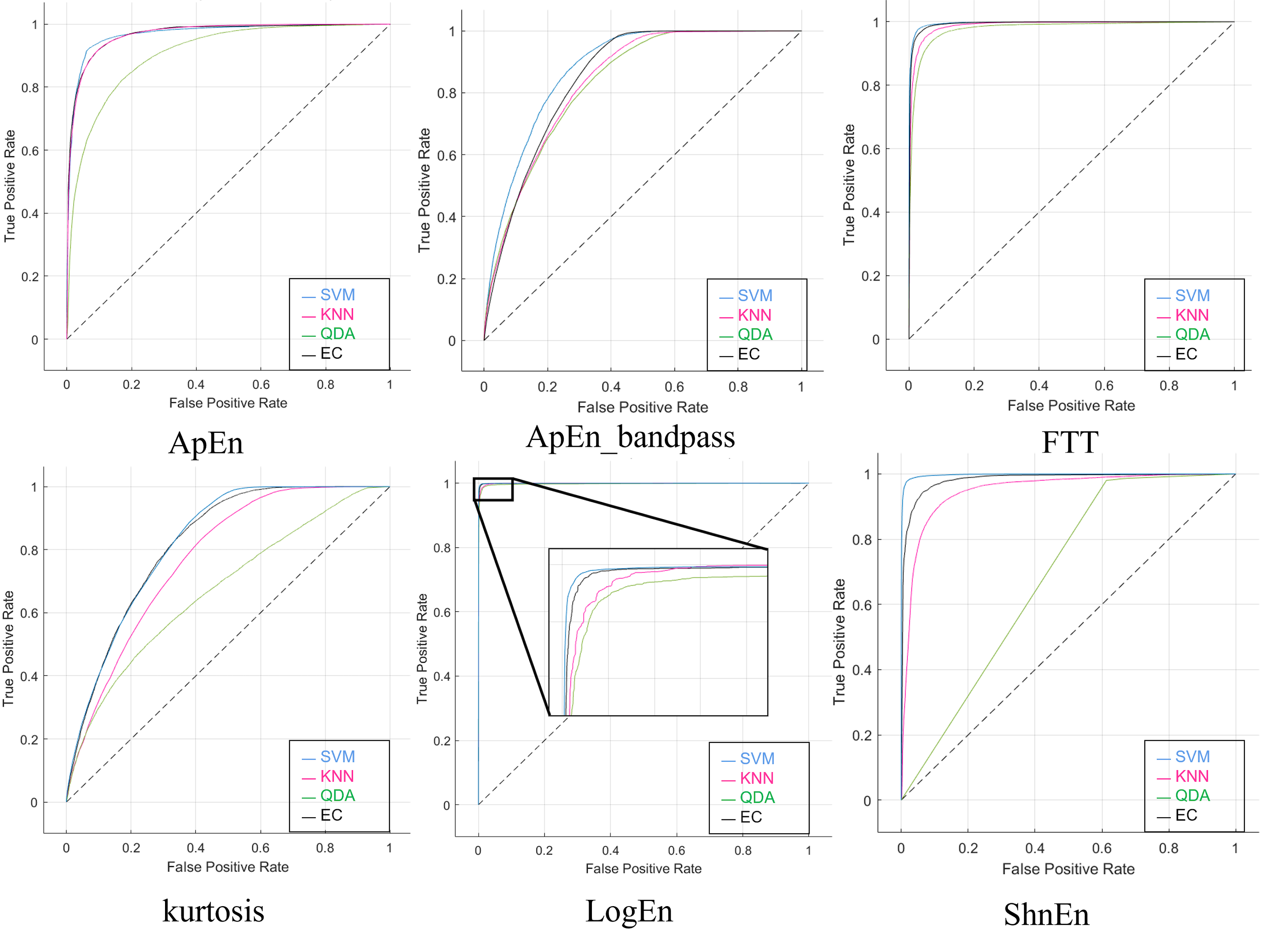

Supplement: Supplemental Information 18 [file peerj-cs-10-2170-s018.png]

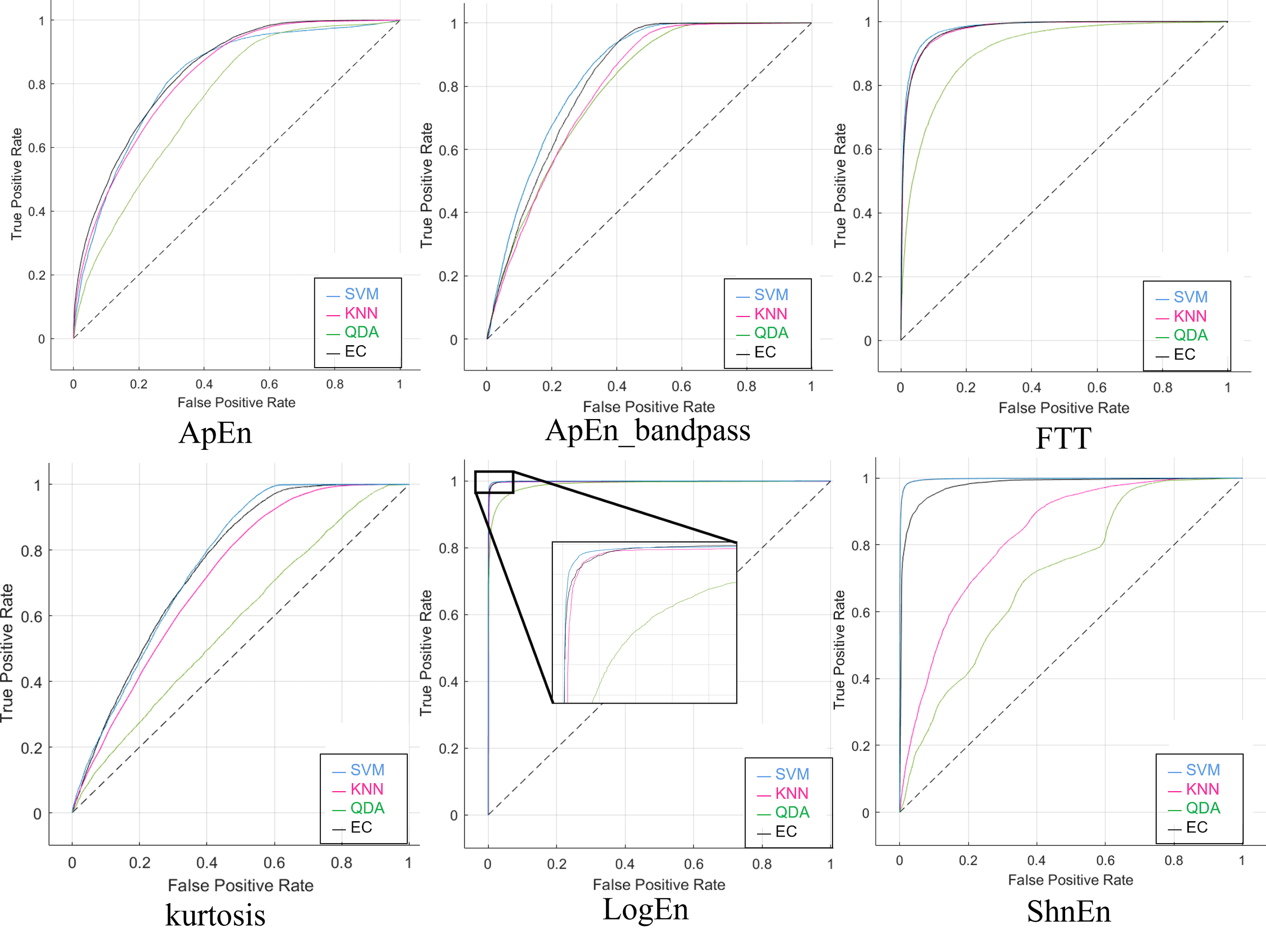

Supplement: Supplemental Information 19 [file peerj-cs-10-2170-s019.png]

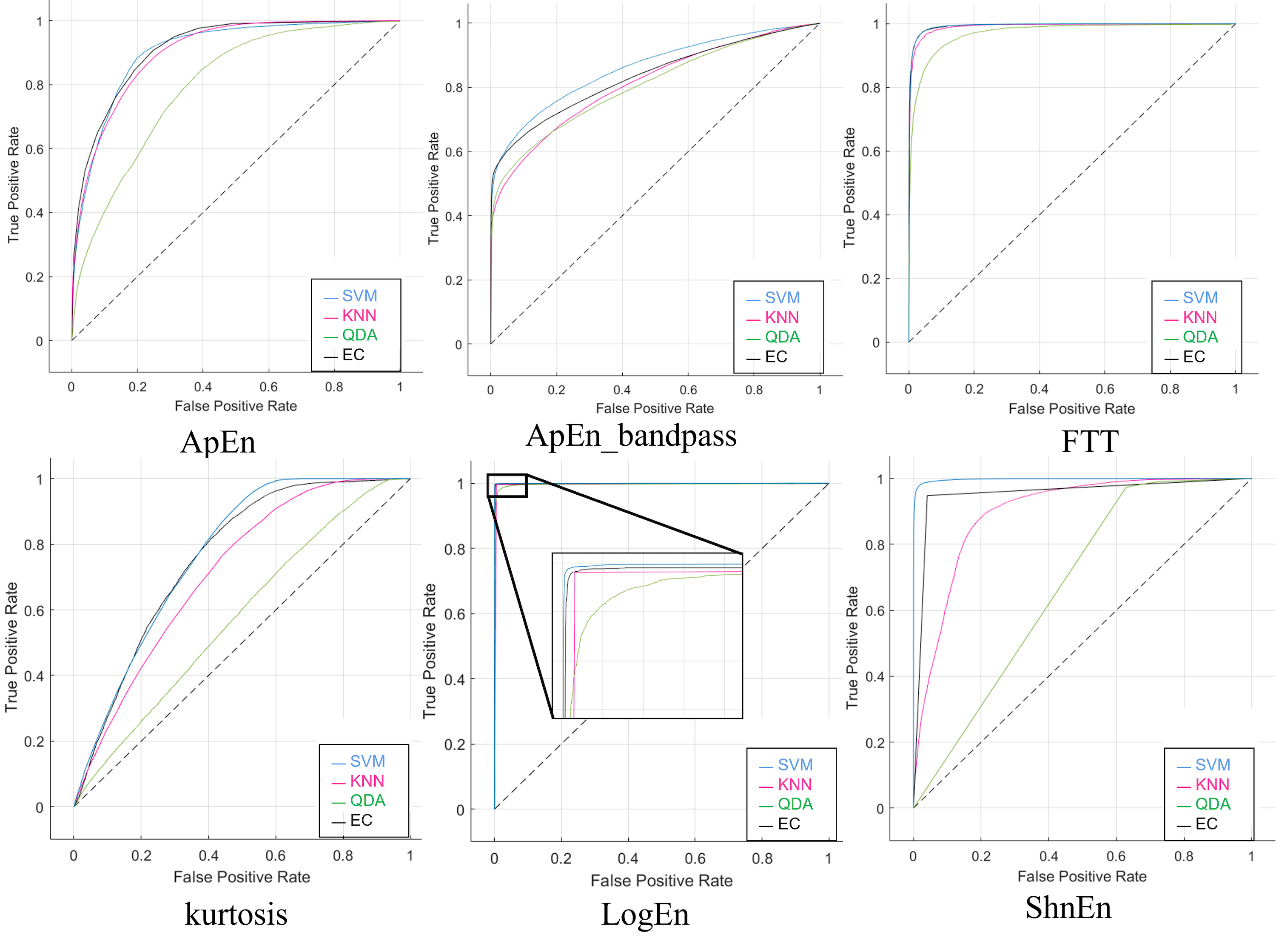

Supplement: Supplemental Information 20 [file peerj-cs-10-2170-s020.png]

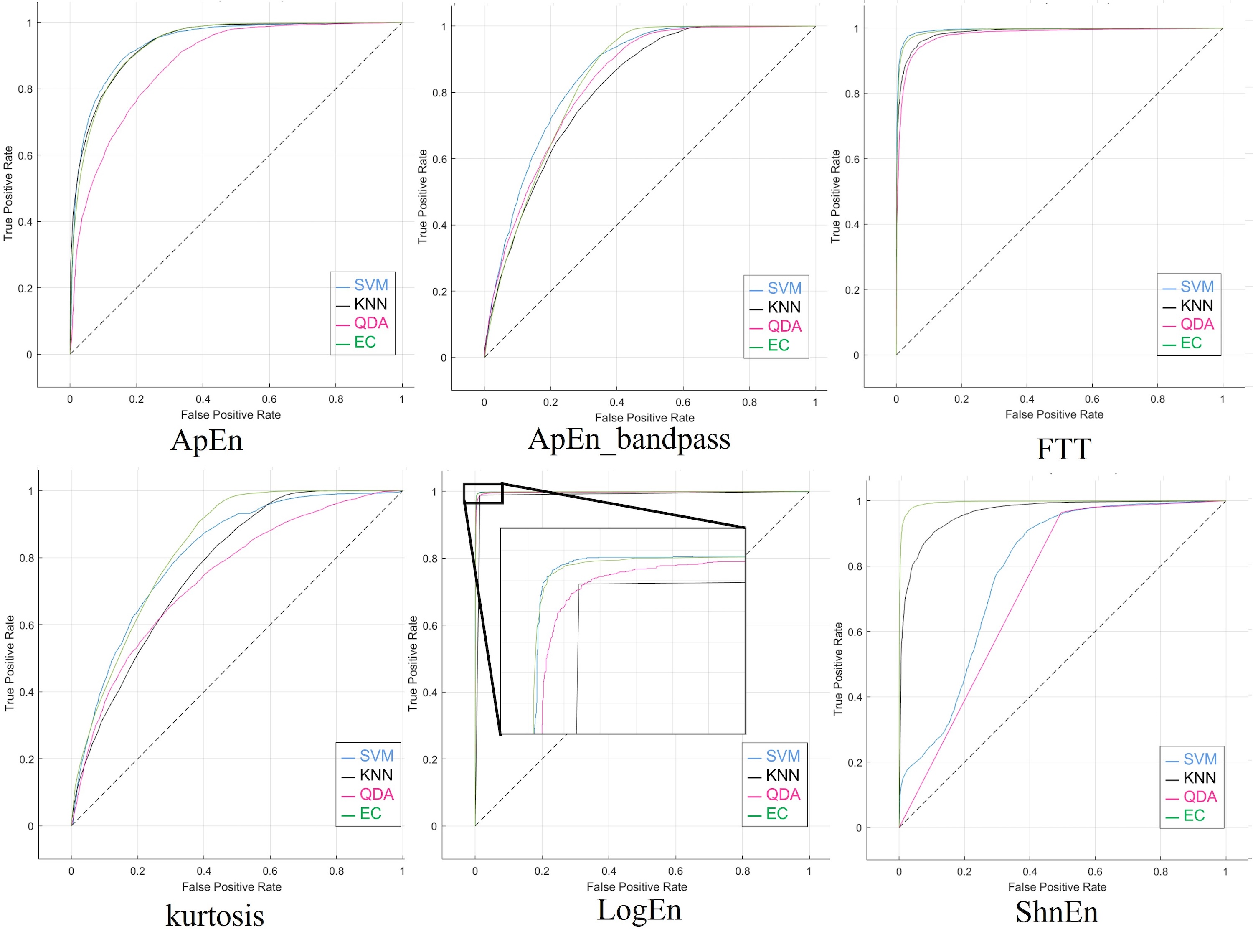

Supplement: Supplemental Information 21 [file peerj-cs-10-2170-s021.jpg]

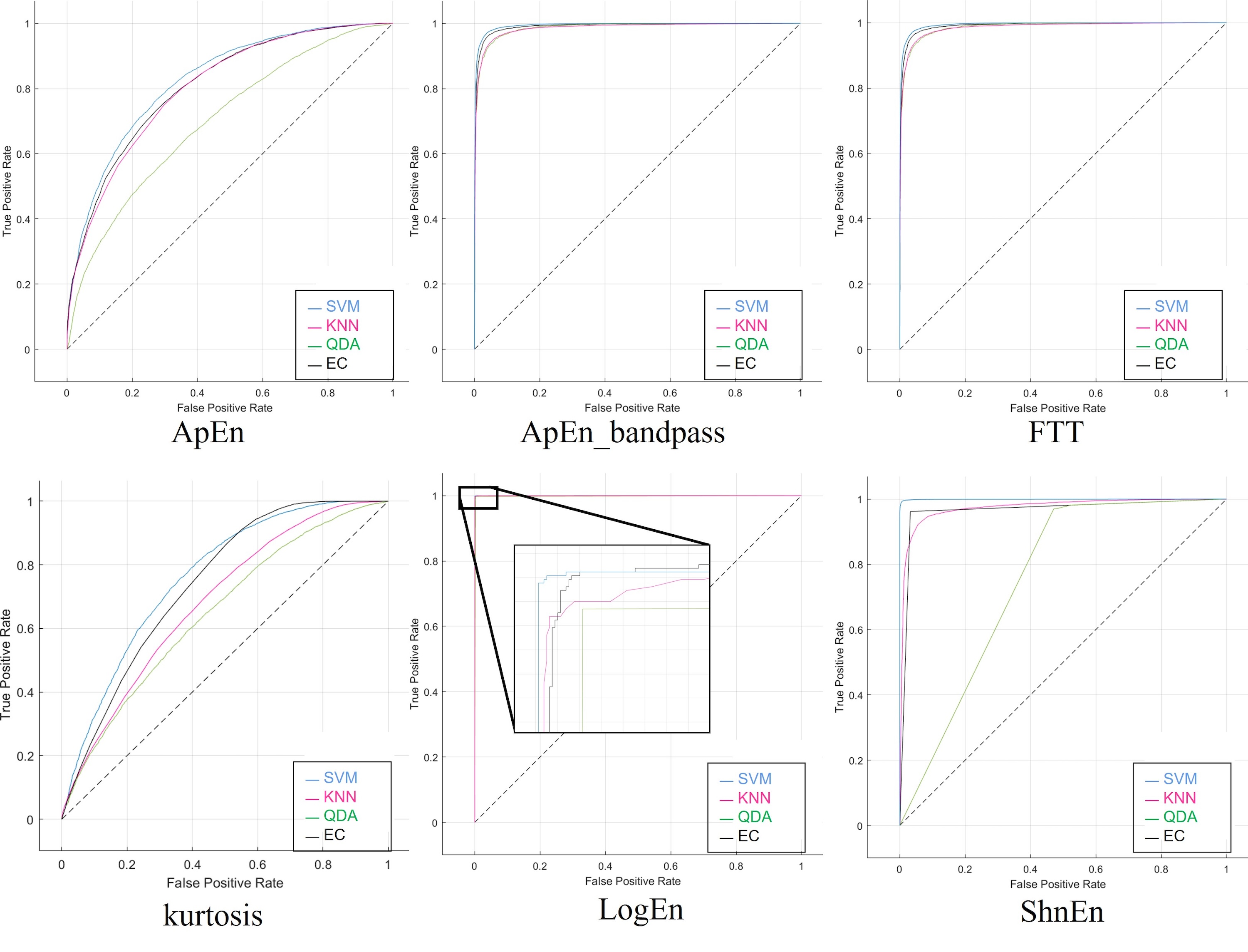

Supplement: Supplemental Information 22 [file peerj-cs-10-2170-s022.jpg]

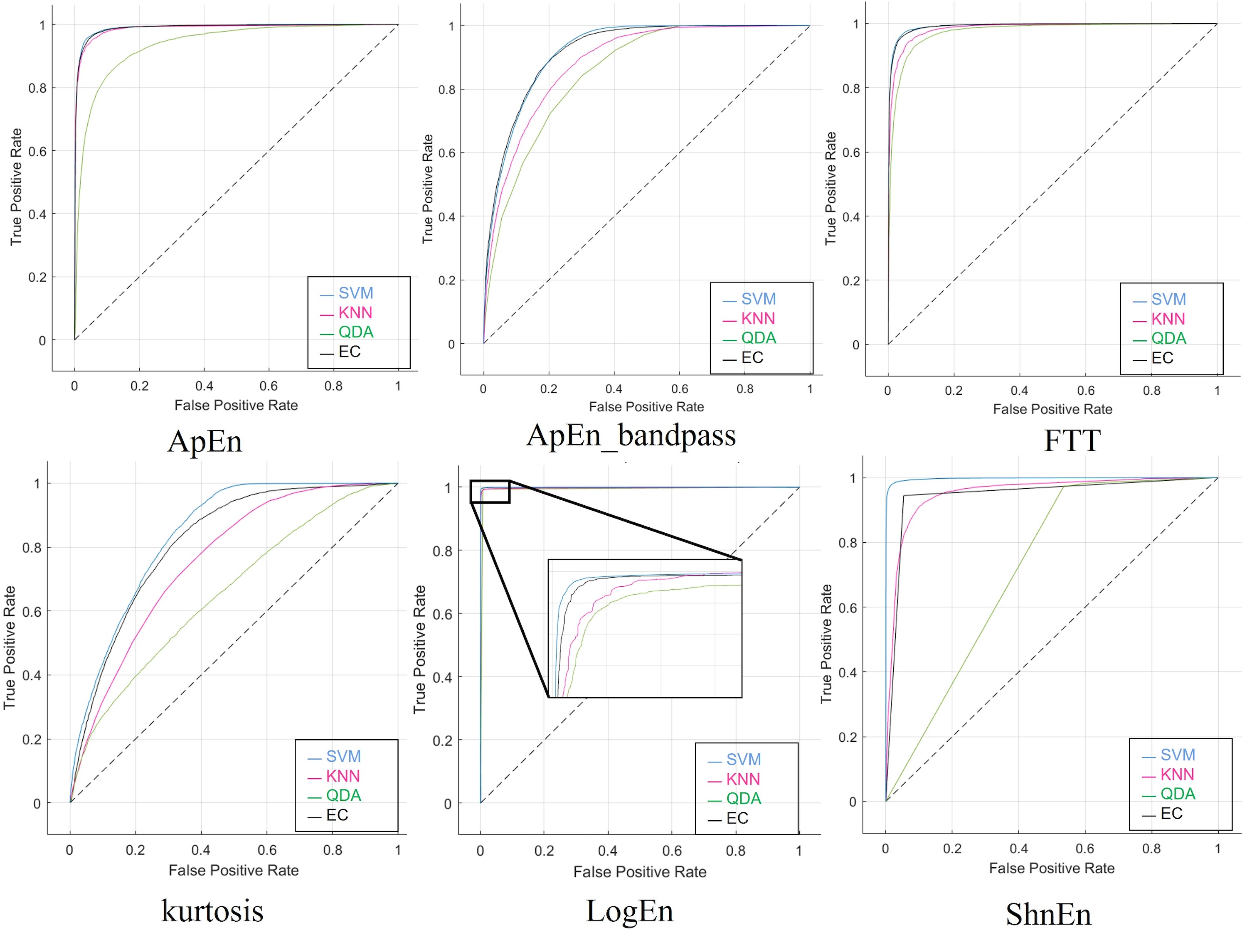

Supplement: Supplemental Information 23 [file peerj-cs-10-2170-s023.jpg]

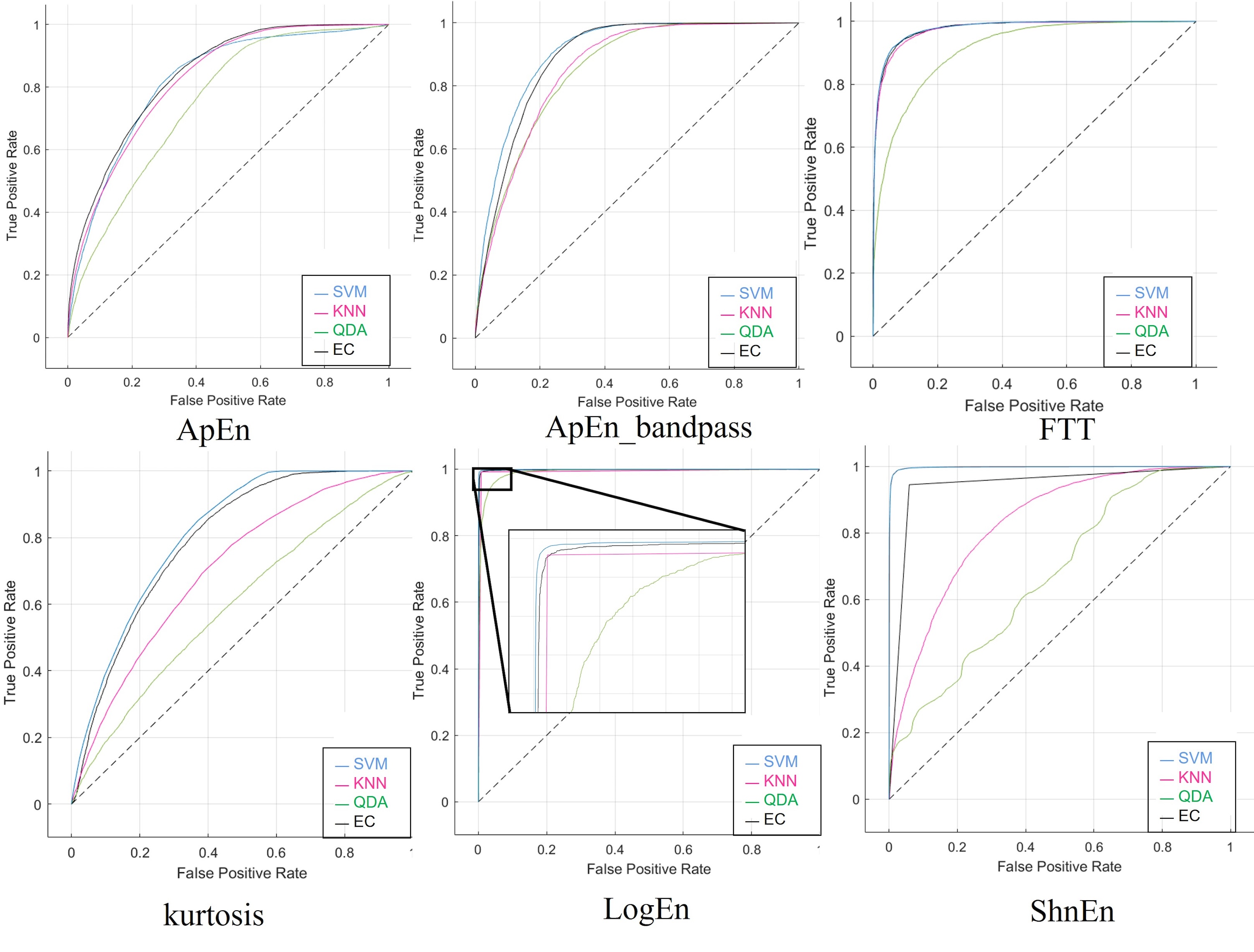

Supplement: Supplemental Information 24 [file peerj-cs-10-2170-s024.jpg]

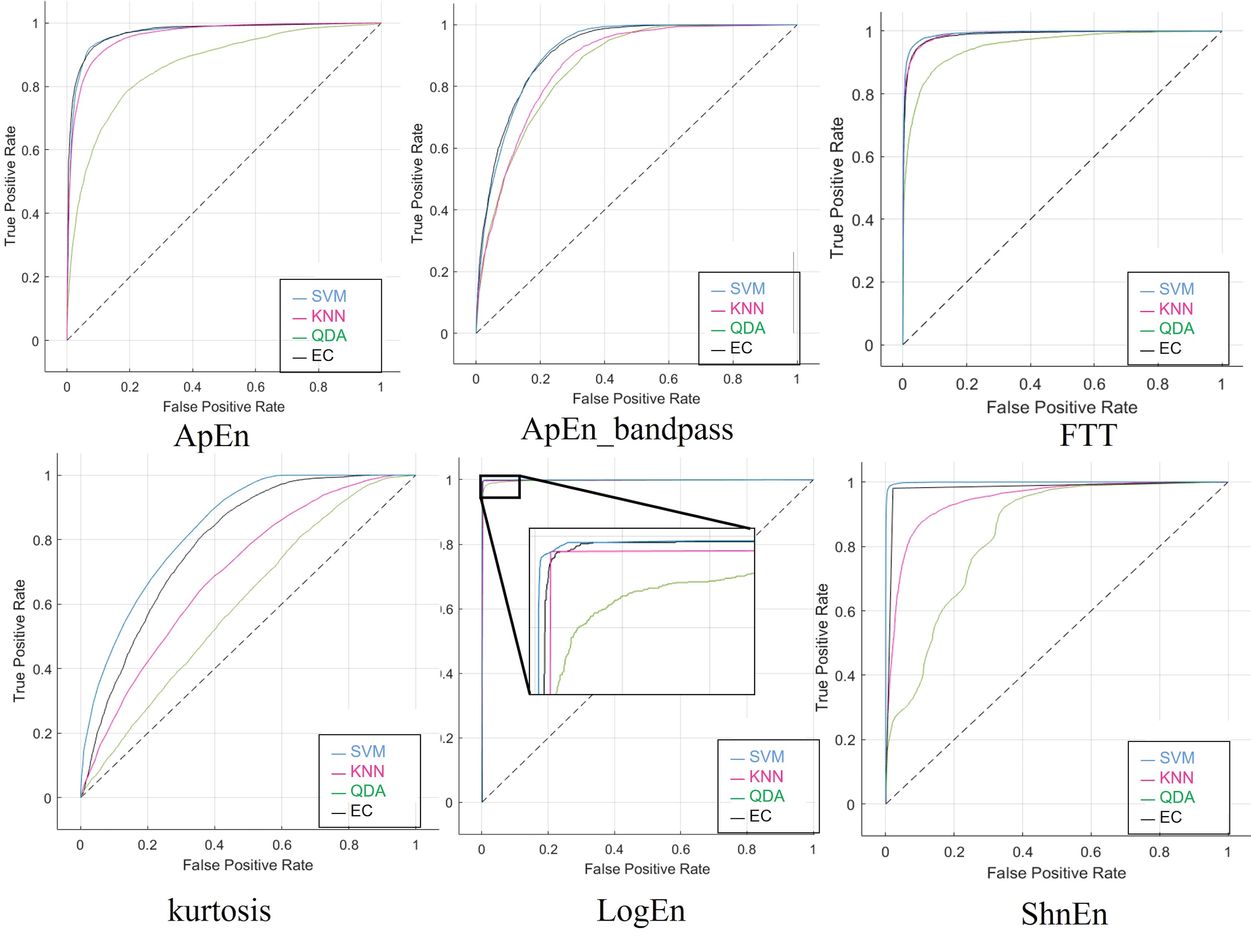

Supplement: Supplemental Information 25 [file peerj-cs-10-2170-s025.jpg]

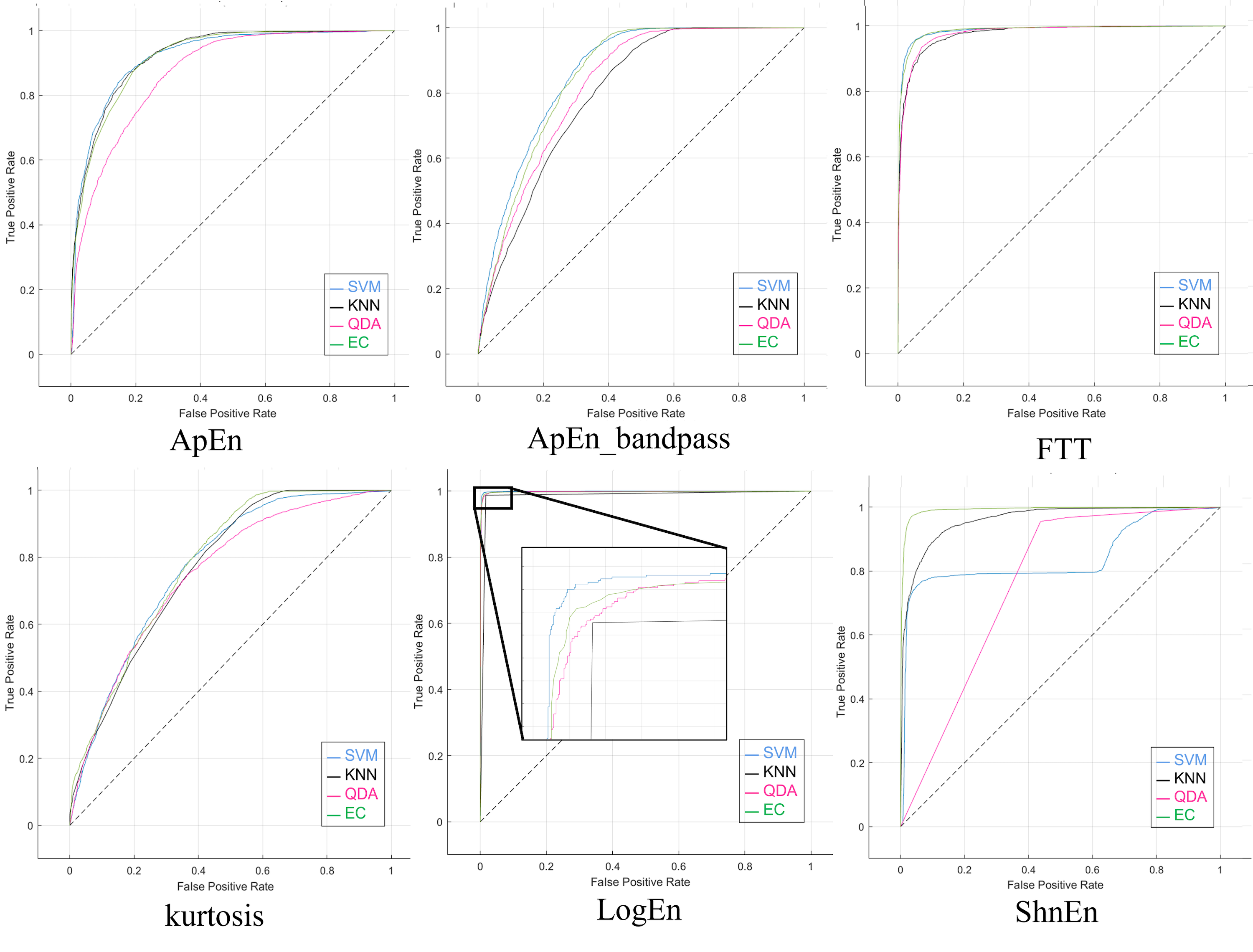

Supplement: Supplemental Information 26 [file peerj-cs-10-2170-s026.png]

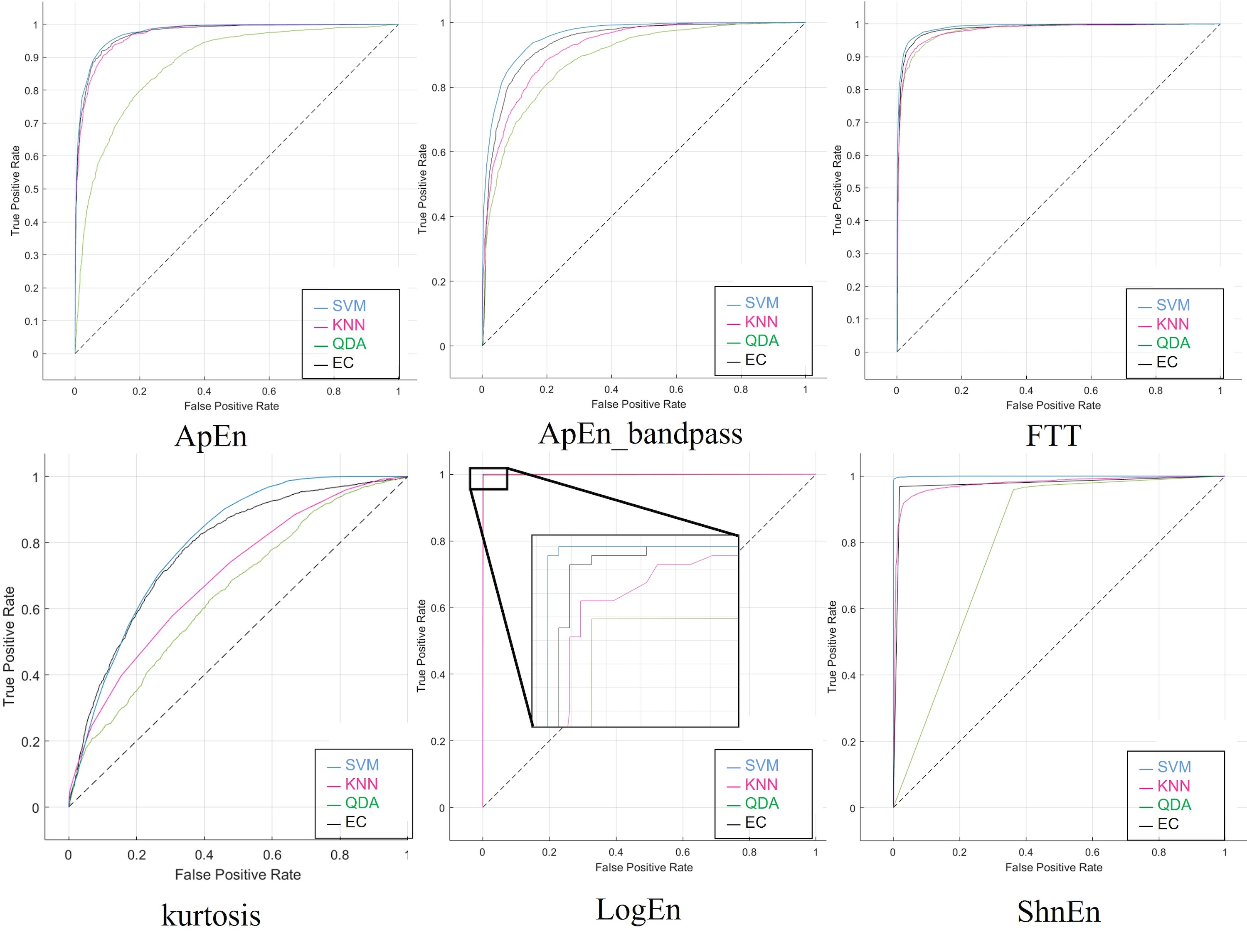

Supplement: Supplemental Information 27 [file peerj-cs-10-2170-s027.jpg]

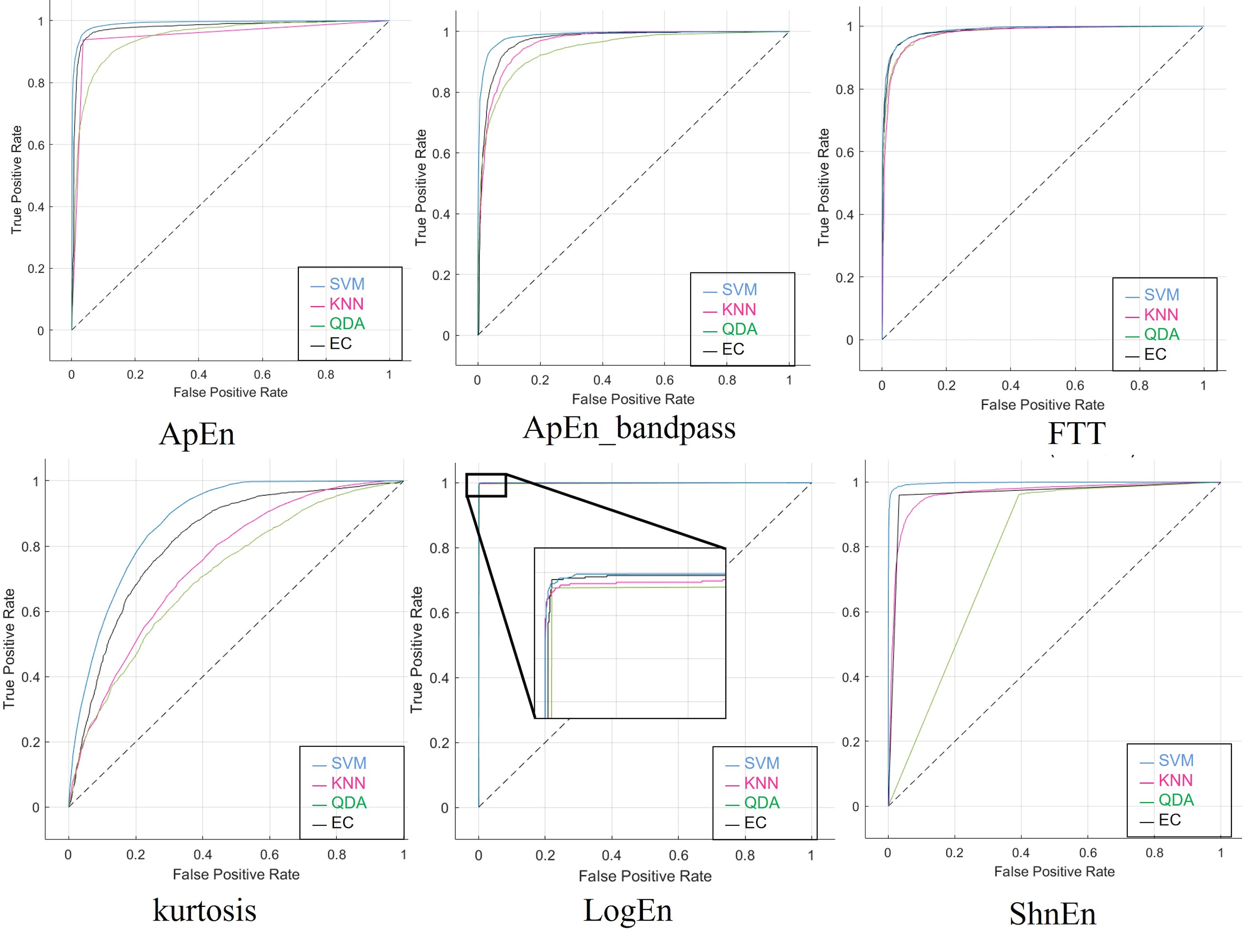

Supplement: Supplemental Information 28 [file peerj-cs-10-2170-s028.jpg]

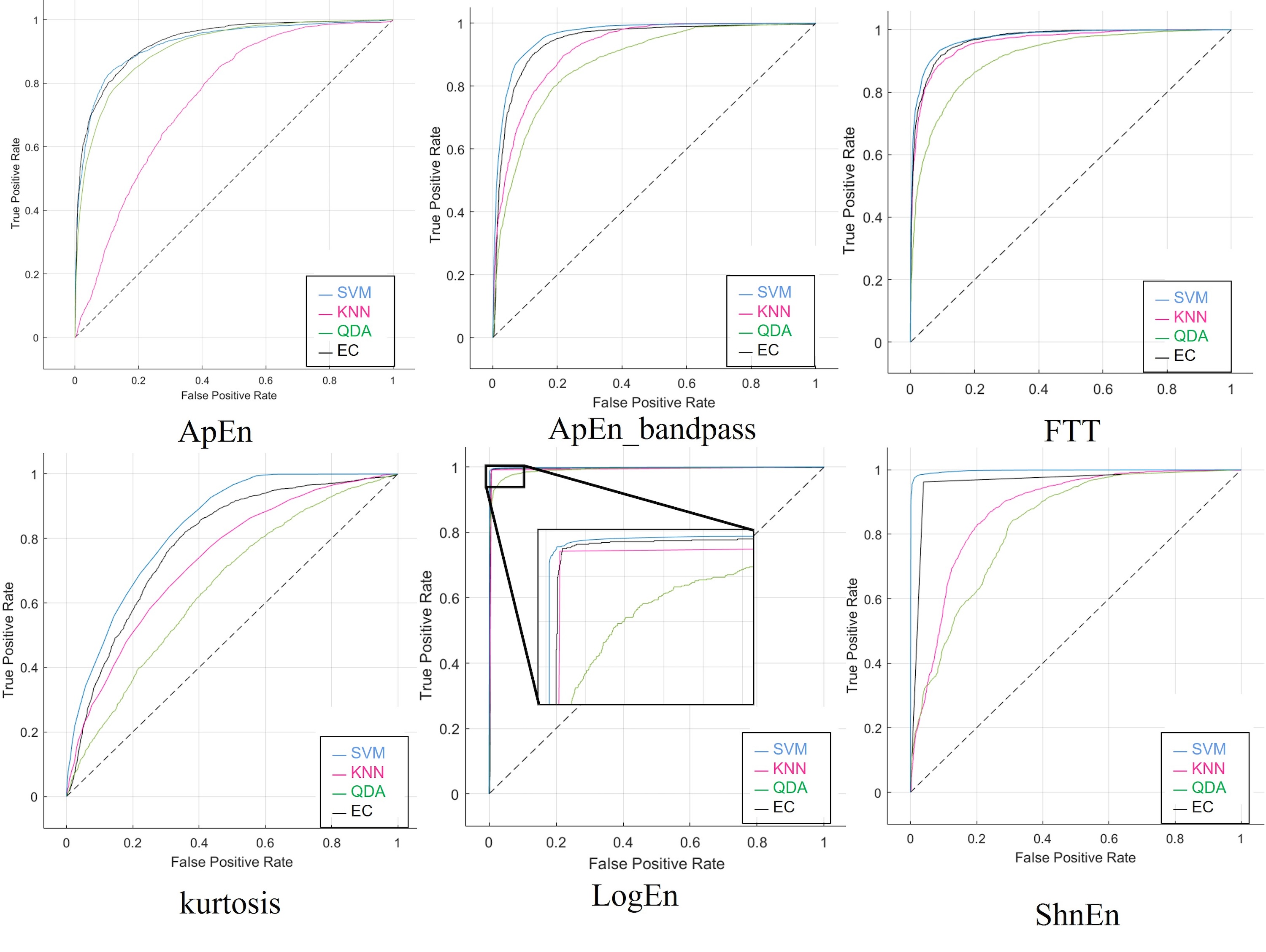

Supplement: Supplemental Information 29 [file peerj-cs-10-2170-s029.jpg]

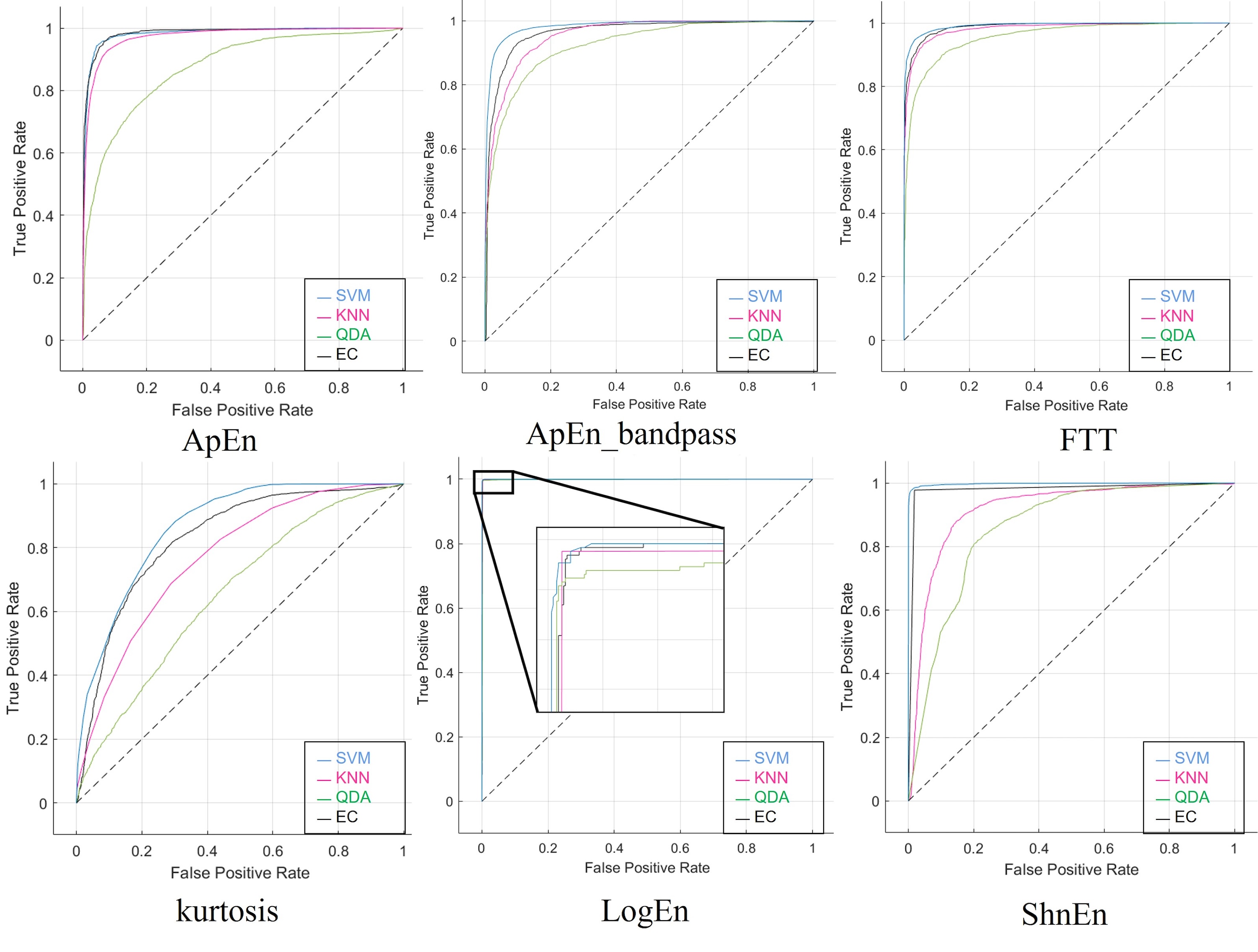

Supplement: Supplemental Information 30 [file peerj-cs-10-2170-s030.jpg]
